# The Melanocortin System: A Promising Target for the Development of New Antidepressant Drugs

**DOI:** 10.3390/ijms24076664

**Published:** 2023-04-03

**Authors:** Dmitrii D. Markov, Oleg V. Dolotov, Igor A. Grivennikov

**Affiliations:** 1 National Research Center “Kurchatov Institute”, Kurchatov Sq. 2, 123182 Moscow, Russia; molgenebio@gmail.com (D.D.M.); olegd@img.ras.ru (O.V.D.); 2 Faculty of Biology, Lomonosov Moscow State University, Leninskie Gory, 119234 Moscow, Russia

**Keywords:** melanocortins, depression, inflammation, hypothalamic-pituitary-adrenal axis, neurotrophins, neurogenesis, brain-derived neurotrophic factor, antidepressants

## Abstract

Major depression is one of the most prevalent mental disorders, causing significant human suffering and socioeconomic loss. Since conventional antidepressants are not sufficiently effective, there is an urgent need to develop new antidepressant medications. Despite marked advances in the neurobiology of depression, the etiology and pathophysiology of this disease remain poorly understood. Classical and newer hypotheses of depression suggest that an imbalance of brain monoamines, dysregulation of the hypothalamic-pituitary-adrenal axis (HPAA) and immune system, or impaired hippocampal neurogenesis and neurotrophic factors pathways are cause of depression. It is assumed that conventional antidepressants improve these closely related disturbances. The purpose of this review was to discuss the possibility of affecting these disturbances by targeting the melanocortin system, which includes adrenocorticotropic hormone-activated receptors and their peptide ligands (melanocortins). The melanocortin system is involved in the regulation of various processes in the brain and periphery. Melanocortins, including peripherally administered non-corticotropic agonists, regulate HPAA activity, exhibit anti-inflammatory effects, stimulate the levels of neurotrophic factors, and enhance hippocampal neurogenesis and neurotransmission. Therefore, endogenous melanocortins and their analogs are able to complexly affect the functioning of those body’s systems that are closely related to depression and the effects of antidepressants, thereby demonstrating a promising antidepressant potential.

## 1. Introduction

Major depressive disorder (MDD) is one of the most common mental disorders. According to the World Health Organization, around 280 million people worldwide suffer from depression [1]. Only a small proportion of people suffering from depression use mental health services. In high-income countries, 33% of people with symptoms of depression use mental health services, and only 8% in low-income countries. Even fewer patients receive minimally adequate treatment (23% in high-income countries and 3% in low-income countries) [2].

According to the Diagnostic and Statistical Manual of Mental Disorders (DSM-5), to be diagnosed with Major Depressive Disorder, a person must have at least 5 of the following 9 symptoms for at least two weeks: depressed mood, markedly diminished interest or pleasure in all or almost all activities (anhedonia), decrease or increase in appetite (weight loss or weight gain), insomnia or hypersomnia, psychomotor agitation or retardation, fatigue or loss of energy, feelings of worthlessness or excessive or inappropriate guilt, diminished ability to think or concentrate or indecisiveness, recurrent thoughts of death (suicidal ideation) [3]. The patient must necessarily have at least one core symptom: depressed mood or anhedonia. Obviously, patients with MDD will be characterized by significantly different combinations of symptoms, and two patients with the same diagnosis may have only one symptom in common [4]. Such a variety of symptoms of MDD suggests the involvement of various brain systems in the manifestation of this disease.

Despite significant advances in the study of depression in recent decades, the mechanisms of the onset and development of depression remain poorly understood. The mechanisms of the therapeutic effects of pharmacological agents used to treat depression are also obscure.

The heritability of depression is estimated at 31–42% [5,6]. Despite the identification of genetic loci thought to increase the risk of developing depression, genome-wide association studies have not led to the discovery of genes associated with the development of this mental disorder [7,8,9,10]. Transcriptome-wide association studies indicate changes in the expression of a large number of genes in depression [11,12,13]. But the reproducibility of both transcriptome-wide association studies and genome-wide association studies is still low. Such unsuccessful attempts to identify genes are explained by the polygenic nature of depression, when the contribution of each individual gene to the development of depression is small. Attempts are also being made to detect genes associated with the development of depression and associated with dysfunction of various physiological systems. For this purpose, gene expression is assessed in post-mortem brain samples and peripheral blood of depressed patients. Such studies have led to the discovery of potential candidate genes involved in inflammation, neuroplasticity, synaptic transmission, and HPAA regulation [14,15,16,17].

Depression is a complex heterogeneous disease that depends on a combination of genetic and environmental factors. The most influential hypotheses of depression are as follows: monoamine (assumes a decrease in monoamine levels as a cause of depression), neurotrophic (a decrease in neurotrophic factors levels, mainly BDNF), impaired neurogenesis in the hippocampus, neuroendocrine (hyperactivation of the HPAA), glutamate (altered glutamatergic excitation), and immune/inflammatory (increased levels of inflammatory cytokines) hypotheses [18,19,20,21]. Probably, all these hypotheses reflect various interrelated aspects of the pathogenesis and manifestation of depression and/or correspond to different processes leading ultimately to the onset of this disease.

Most of the current drug treatments for depression are based on the monoamine hypothesis, which considers a decrease in brain levels of monoamines as the cause of the development of depressive symptoms. First-generation antidepressants include tricyclic antidepressants and monoamine oxidase inhibitors, while better-tolerated second-generation antidepressants include selective serotonin reuptake inhibitors (SSRIs), serotonin and norepinephrine reuptake inhibitors (SNRIs), and norepinephrine and dopamine reuptake inhibitors (NDRIs) [22,23]. Both first- and second-generation antidepressants have a number of adverse side effects. Among the most common undesired effects are nausea, diarrhea, weight gain, drowsiness, insomnia, dizziness, headache, and sexual dysfunction. The most dangerous side effect of antidepressants is an increased risk of suicide in depressed children and young adults [24]. Side effects of antidepressants, which are often intolerable, limit their use in clinical practice. Almost 40% of patients do not experience long-term improvement after antidepressant treatment. Such cases are referred to as a treatment-resistant depression (TRD)—the impossibility of achieving and maintaining euthymia during therapy with various types of antidepressants [25,26]. The resistance of a significant proportion of patients to antidepressants may also indicate the existence of different mechanisms underlying depression onset in different patients. 

In addition to side effects and resistance to antidepressants, another significant drawback is the need for their long-term use to achieve a therapeutic effect. The effectiveness of clinically used antidepressants is also questionable [27,28]. The monoamine hypothesis of depression is firmly entrenched not only in public consciousness, but also in the scientific community and the strategies of pharmaceutical companies. However, the monoamine hypothesis is being seriously criticized, in part, due to the fact that convincing evidence indicating the involvement of serotonin in the development of depression has not been presented [29].

Because of the obvious shortcomings of antidepressants used in clinical practice, there is a need to find alternative approaches to the treatment of this mental disorder. There is an urgent need for antidepressant drugs that are effective in patients with TRD, are better tolerated by patients, and have a faster therapeutic effect. As noted above, traditional antidepressants demonstrate a delayed therapeutic effect. At the same time, the development of fast-acting antidepressants is principally possible, as evidenced by the effectiveness of sleep deprivation and the effects of low doses of ketamine [30]. In the case of ketamine, it has been shown that its administration leads to an improvement in symptoms within a few hours [31,32,33]. The mechanisms of the antidepressant effects of this noncompetitive NMDA receptor antagonist are still unclear. However, the recently demonstrated involvement of opioid system activation in the antidepressant effects of ketamine [34,35] not only may be related to its rapid antidepressant effects, but also raises questions about its safety in long-term use.

Peptidergic systems may be potential targets for the development of drugs for the treatment of depression. It is assumed that stress-related neuropeptides may play an important role in the development of anxiety and depression [36], while both the neuropeptides themselves and their receptors are considered as potential targets for the treatment of mental disorders [37,38,39].

One such system is the melanocortin system, consisting of adrenocorticotropic hormone (ACTH)-activated receptors and their ligands (melanocortins). ACTH is a key component of the body’s stress response. Melanocortin receptors mediate various effects of ACTH and related peptides in the brain and periphery. Interest in the study of the central melanocortin system was significantly stimulated by its critical involvement in the regulation of energy balance and body weight [40]. There are very close interrelationships at the functional and neuroanatomical levels between the regulation of energy balance and the neuroendocrine stress response, and this is confirmed by the high comorbidity of obesity-related pathologies and stress-related mental disorders [41]. The melanocortin system is a critical component and regulator of the neuroendocrine stress response, and its role in stress and stress-induced pathologies, such as anxiety and depression, is also being actively studied [42].

The aim of this review is to discuss the currently known biological activities of melanocortins in the context of the hypotheses of depression, and to consider the properties of the peptide family of melanocortins from the perspective of the development of new pharmacological antidepressant treatments.

## 2. The Melanocortin System

The melanocortin system consists of a family of melanocortin peptides and a family of their receptors [43]. Melanocortins are a family of peptides derived from the 26 kDa proopiomelanocortin (POMC) precursor. POMC processing results in a number of bioactive peptides, including ACTH, α-melanocyte stimulating hormone (α-MSH), β-melanocyte stimulating hormone (β-MSH), γ-melanocyte stimulating hormone (γ-MSH), β-lipotropic hormone (β-LPH), and β-endorphin [44]. Amino acid sequences of some natural human melanocortins are presented in Table 1.

All melanocortins originate from different parts of proopiomelanocortin molecule by limited proteolysis. α-MSH is the N-terminal part of the ACTH molecule, β-MSH originates from beta-lipotropin, and γ-MSH peptides originate from the N-terminal sequence of proopiomelanocortin. Modifications of N-terminal amino acids (acylation) or amidation of the C-terminal alter the stability and activity of these peptides.

The main source of proopiomelanocortin is the pituitary gland (its anterior and intermediate lobes), however, POMC mRNA is also found in other brain structures, as well as in peripheral organs and tissues, such as lymphocytes, skin, placenta, pancreas, thyroid gland, testes, intestine, kidneys, and liver [45]. α-MSH in the rat brain is also characterized by a scattered distribution. Its highest content was found in the neurons of the arcuate nucleus of the hypothalamus. α-MSH was not found in the cerebral cortex and cerebellum [46].

The physiological effects of melanocortins are mediated through their interaction with melanocortin receptors (MCRs). Cloning of the MCR genes has led to tremendous progress in understanding the biological role of melanocortins. Five subtypes of MCRs have been identified (MC1R, MC2R, MC3R, MC4R, MC5R) [47]. MCRs are classic G-protein coupled receptors with seven transmembrane domains. MCRs have 40–60% amino acid sequence homology, and they differ in their tissue distribution and affinity for various melanocortins and physiological antagonists, such as ASIP (agouti-signaling protein) and AGRP (agouti-related protein) [47,48]. ACTH, a peptide of 39 amino acids residues, and its N-terminal fragments longer than 1–16 activate all five MCR subtypes. α-MSH activates four subtypes (MC1R, MC3R, MC4R, MC5R), however shorter α-MSH fragments are not able to activate MC1R but still can activate other subtypes of MCRs [49]. The expression of MC3R, MC4R, and MC5R subtypes has been found in the brain [49,50]. By binding to the corresponding receptor, melanocortins are able to activate a number of signaling cascades, such as: AC/cAMP/PKA, PLCβ/DAG/PKC, PLCβ/IP3/Ca^2+^, Jak/STAT, PI3K/ERK1/2 [51].

The specific effect exerted by MCR agonists depends on the subtype of the activated receptor and its tissue localization. MC1R is responsible for skin and hair pigmentation, MC2R is required for steroidogenesis in the adrenal cortex, MC3R and MC4R are involved in the control of food intake and behavior, and MC5R plays an important role in sebogenesis [43].

The accessory proteins of the MCRs (MRAP and MRAP2) are also important. These proteins interact with all five MCRs, are involved in the trafficking of receptors from the endoplasmic reticulum to the plasma membrane, modulate their activity upon binding to ligands, and are involved in the internalization of receptors [52,53].

Mutations in the genes of MCRs and accessory proteins can lead to the development of a number of diseases. Mutations in the *MC1R* gene are associated with an increased risk of melanoma, *MC2R* mutations result in familial glucocorticoid deficiency, and mutations in the *MC4R* and *MRAP2* genes are associated with severe forms of obesity [54]. As there is both a wide variety of functions are carried out by the activation of melanocortin receptors, and there are many diseases associated with mutations in the genes of these receptors, they have become attractive targets for drug development.

In addition to endogenous melanocortins, a large number of their analogs have been synthesized [55]. Currently, several melanocortin-based drugs have already been approved for clinical use: Acthar^®^ Gel—full-length ACTH1-39 (treatment of multiple sclerosis and infantile spasms), Cortrosyn^TM^—an ACTH1-24 fragment (used to diagnose adrenal insufficiency), Synacthen ^®^Depot—a fragment of ACTH1-24 (treatment of multiple sclerosis, rheumatoid diseases, ulcerative colitis, nephrotic syndrome, and as a diagnostic test for adrenal insufficiency), Scenesse^®^—Afamelanotide, an α-MSH analogue (treatment of erythropoietic protoporphyria), Vyleesi^®^—Bremelanotide, a cyclic heptapeptide (treatment of hypoactive sexual desire disorder in women), and Imcivree ^®^—Setmelanotide, a cyclic octapeptide (treatment of monogenic or syndromic obesity) [56].

## 3. The Monoamine Hypothesis of Depression and Melanocortins

The monoamine hypothesis of depression suggests that depression is associated with a deficiency or imbalance of monoamine neurotransmitters, such as serotonin, norepinephrine, and dopamine. Although this hypothesis is based on the fact that various antidepressants increase brain monoamine levels by inhibiting their metabolism or reuptake, there is no evidence that a decrease in brain monoamine levels can cause depression. Monoamine depletion studies demonstrated decreased mood in subjects with a family history of MDD and in drug-free patients with MDD in remission, but not in healthy humans [57]. The depressogenic activity of reserpine, an alkaloid that irreversibly blocks vesicular monoamine transporters and reduces monoamine levels in the synaptic cleft, has long been accepted as evidence of a causal relationship between low monoamine levels and depression. However, there are no firm evidences that reserpine is depressogenic [58,59]. On the other hand, monoamine deficiency or imbalance may reflect complex brain dysfunctions associated with depression, and the therapeutic effectiveness of antidepressants may be due to the complex normalization of these disturbances, the mechanism of which includes an antidepressant-induced increase in monoamine levels. Accumulating evidence suggests this complexity, such as the interconnected roles of serotonin, cytokines, and neurotrophins in depression and antidepressant therapy [60]. 

In vivo studies indicate that melanocortins are able to influence brain monoamine levels, both after central and peripheral administration. The ability of these peptides after central administration to influence grooming behavior, locomotor activity, and reward behavior, indicate a close relationship between the melanocortin and dopaminergic systems [61]. Most studies indicate an increase in dopamine turnover after central melanocortin administration, but the cellular and molecular mechanisms of this effect are not known [62,63]. α-MSH administration into the ventral tegmental area (VTA), which together with the nucleus accumbens (NAc) forms the brain reward/aversive system, induce a significant increase in dopamine levels in the NAc. This increase is completely blocked by pre-treatment with the MC4R selective antagonist HS131 [64]. Centrally administered melanocortins may affect not only dopamine release, but also dopamine receptors. Melanocortin receptor agonist melanotan-II (MT-II) induces changes in dopamine D1-like and D2-like receptor binding in several regions of the rat brain [65]. ACTH1-24 inhibits association and dissociation of the dopamine D2 agonist to the dopamine D2 receptor, suggesting a binding site for ACTH1-24 on the D2 receptor [66].

There are no direct data on the effects of centrally administered melanocortin receptor agonists and antagonists on norepinephrine and serotonin levels. However, intracerebroventricular (ICV) injection of MT-II reduces the firing rate of locus coeruleus noradrenergic neurons and increases the firing rate of dorsal raphe nucleus serotonergic neurons [67]. Peripherally administered non-corticotropic melanocortins may affect brain norepinephrine and serotonin levels. Subchronic peripheral ACTH4-10 administration results in an increase of tyrosine hydroxylase activity in the locus coeruleus, a main brain source of norepinephrine [68]. These data are consistent with the observed increase in the rate of catecholamine synthesis in the whole brain and brain stem of intact rats following subcutaneous (SC) administration of ACTH4-10 [69]. SC pre-treatment of rats with α-MSH inhibits a stress-induced increase in cortical serotonin reuptake levels [70], suggesting that peripheral melanocortins may directly or indirectly regulate serotonin levels in the synaptic cleft under stress conditions. Systemic administration of Semax (Met-Glu-His-Phe-Pro-Gly-Pro), an ACTH4-10 analog, failed to alter striatal concentrations of dopamine and its metabolites but significantly increased the levels of serotonin and its metabolite 5-hydroxyindoleacetic acid [71].

Collectively, these data indicate that central melanocortin agonists can act as regulators of the dopaminergic system, increasing its activity and affecting the reward/aversion circuit. Peripherally administered melanocortin agonists can activate the serotonergic and noradrenergic systems in intact and stress-exposed animals. Although there is only fragmentary data on this ability, the reported effects of melanocortins suggest that it is consistent with the hypothesis that such activity is related to the therapeutic effects of antidepressant drugs. The effects of melanocortins on the dopaminergic, serotonergic, and noradrenergic systems are summarized in Table 2.

## 4. The Inflammatory Hypothesis of Depression and Anti-Inflammatory Effects of Melanocortins

The inflammatory hypothesis of depression is based on the following main observations: (1) increased levels of pro-inflammatory cytokines in depressed patients, (2) depressive symptoms in patients suffering from inflammatory diseases, and (3) an increased risk of depression in patients undergoing pro-inflammatory cytokine therapy [72,73,74]. There is a vast body of evidence showing elevated levels of various pro-inflammatory cytokines (IL-1β, IL-6, TNF-α) in the blood of depressed patients [75,76,77]. The important role of pro-inflammatory cytokines is evidenced by the fact that IFN-α, used to treat a number of diseases, often causes symptoms of depression [78]. 

Studies using experimental endotoxemia in humans [79] show that intravenous administration of lipopolysaccharide (LPS) (0.4–0.8 ng/kg) induces depressed moods, increases the level of anxiety [80,81,82], leads to the development of sickness and depressive symptoms, and suppresses motivational behavior [83]. Along with mood changes, an increase in the level of pro-inflammatory cytokines [84,85], activation of HPAA, which is confirmed by a significant increase in the level of cortisol [86], and an increase in the norepinephrine level [87,88] is observed in humans.

Depression can hardly be considered as an inflammatory disease. The inflammatory process is neither necessary nor sufficient for the onset of depression. But activation of the immune system can disrupt the functioning of the nervous and neuroendocrine systems [89]. What the cause is of a low-grade inflammatory process in depression is not clear. Factors associated with the development of systemic inflammation and an increased risk of depression include psychosocial stressors, poor diet, physical inactivity, obesity, smoking, altered gut permeability, atopy, sleep disturbances, and vitamin D deficiency [90]. Not all studies support an association between elevated cytokine levels and depression [91]. Pro-inflammatory cytokine levels in healthy individuals and depressed patients may overlap to a large extent. The discriminating ability of cytokine concentration is extremely low, and the changes in their levels is largely non-specific [92].

It is known that antidepressants used to treat depression have immunomodulatory properties [93]. Different types of antidepressants are able to reduce the level of cytokines in experimental models in vitro and in vivo, as well as to normalize the level of pro-inflammatory cytokines in depressed patients [94,95,96,97,98,99,100]. Anti-inflammatory treatment decreases depressive symptoms [101]. However, the efficacy of non-steroidal anti-inflammatory drugs (NSAIDs) on depressive symptoms appears negligible [102,103]. Combination therapy is expected to be more effective, although there is evidence of antagonistic effects of NSAIDs and SSRIs in mice and humans [104]. A more recent meta-analysis suggests that anti-inflammatory drugs (NSAIDs, cytokine inhibitors, statins, glucocorticoids, and minocycline) when used in a combination with antidepressants improve antidepressant therapy [105]. It is clear that there is a bidirectional relationship between the immune system and the HPAA, as well as a link between the inflammatory and neuroendocrine hypotheses of depression [106,107]. Chronic hyperactivation of the HPAA, induced by chronic psychosocial stress, may cause glucocorticoid resistance and impaired glucocorticoid inhibition of the inflammatory response and HPAA activity [106], which may explain the elevated levels of pro-inflammatory cytokines accompanied by elevated cortisol levels in a subset of depressed patients. Chronic inflammation can chronically activate the HPAA. The weak antidepressant efficacy of NSAIDs may be due to the lack of ability to affect other disturbances associated with depression and normalize HPAA activity. On the contrary, antidepressants exhibit not only anti-inflammatory effects (including direct effects, as follows from their anti-inflammatory activity in vitro), but are also able to restore sensitivity to glucocorticoids and normalize HPAA activity [106]. The increase in the effectiveness of combined antidepressant and anti-inflammatory therapy may indicate the need for a combination of anti-inflammatory activities and the ability to normalize HPAA activity in potential antidepressant drugs.

**Figure 1:**
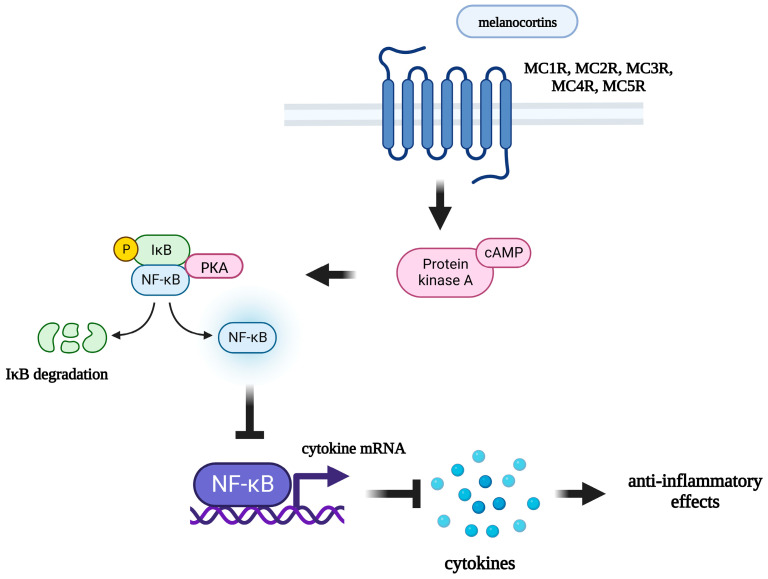
Mechanism of anti-inflammatory effects of melanocortins. MC1R: melanocortin 1 receptor; MC2R: melanocortin 2 receptor; MC3R: melanocortin 3 receptor; MC4R: melanocortin 4 receptor; MC5R: melanocortin 5 receptor; cAMP: cyclic adenosine monophosphate; PKA: protein kinase A; IκB: inhibitor of nuclear factor kappa B; NF-κB: nuclear factor kappa-light-chain-enhancer of activated B cells.

Quite a lot of information has been accumulated that indicates the anti-inflammatory effects of melanocortins. α-MSH is known to exhibit antipyretic, antimicrobial, anti-inflammatory, and immunomodulatory properties [108]. Numerous in vitro and in vivo studies show that melanocortins exert anti-inflammatory effects in a glucocorticoid-dependent (in the case of ACTH) and glucocorticoid-independent manner [108]. α-MSH and related peptides inhibit the secretion of pro-inflammatory cytokines (Figure 1), induce the secretion of anti-inflammatory cytokines, inhibit the expression of adhesion molecules, reduce the secretion of other inflammatory mediators (NO, prostaglandins), and are able to modulate the activity of immunocompetent cells [109]. In vivo, melanocortins exhibit antipyretic and anti-inflammatory effects in experimental models of neuroinflammation and systemic inflammation [108,110,111,112]. The important role of melanocortins in the inflammatory response is evidenced by the fact that injection of bacterial endotoxin leads to an increase in the level of endogenous circulating α-MSH in both animals [113] and humans [114]. In addition, intraperitoneal (IP) administration of LPS leads to an increase in POMC mRNA expression in the arcuate nucleus of the hypothalamus [115]. Intravenous (IV) and ICV administration of α-MSH has an antipyretic effect, inhibiting fever induced by the administration of pro-inflammatory cytokines or bacterial endotoxin [116,117]. Central injection of α-MSH analogue NDP-α-MSH also exhibits an antipyretic effect [118]. Probably, the antipyretic effect of α-MSH depends on the activation of MC3R/MC4R, since the antagonist of these receptors (SHU 9119) completely blocks this effect [119,120]. The selective MC4R antagonist HS014 also blocks the antipyretic effect of α-MSH on LPS-induced fever [121].

Circulating α-MSH has been shown to reduce the production of pro-inflammatory cytokines, thereby modulating inflammatory responses within the brain [122]. Central administration of α-MSH reduces LPS-induced COX-2 and iNOS mRNA expression in the hypothalamus [123]. Both peripheral and central administration of α-MSH results in a decrease in circulating and brain TNF-α levels [124]. Peripheral administration of Semax led to a decrease in the pro-inflammatory cytokine mRNA in the brain in an experimental model of ischemic stroke [125,126]. In a model of neonatal hypoxic-ischemic brain injury, intranasal administration of the MC1R agonist BMS-470539 also led to a decrease in the level of pro-inflammatory cytokines [127]. In vitro α-MSH and ACTH1–24 inhibits the production of TNF-α, IL-6, and NO in cultured murine microglial cell lines [128] and also inhibits TNF-α production in human glioma cell lines [129]. The selective MC4R agonist Setmelanotide suppresses neuroinflammation in human astrocytoma cell lines [130].

The mechanism of the anti-inflammatory action of melanocortins is poorly understood. It is assumed that peptides of this family can act both on the periphery, directly affecting immunocompetent cells expressing melanocortin receptors, and centrally, by activating melanocortin receptors in various brain structures, thereby preventing the development of an inflammatory response in the periphery [131]. Through which specific receptor subtype melanocortins exert their anti-inflammatory effects is currently unknown. It has been shown that all 5 subtypes of MCRs are expressed by immunocompetent cells. MC1R is expressed by macrophages [132], B-lymphocytes, natural killer cells, cytotoxic T-lymphocytes [133], neutrophils [134], and dendritic cells [135]; MC2R has been found on B- and T-lymphocytes, as well as on macrophages [136]; MC3R is expressed by peritoneal macrophages [137]; and MC5R was found on the surface of mouse B-lymphocytes [138]. Expression of all five subtypes of MCRs was also found on human lymphocytes and monocytes [139]. Immunocompetent cells express different subtypes of receptors, but researchers assign the main role in the mediating of anti-inflammatory effects of melanocortins to MC3R and MC4R [140], which are widely expressed in various brain structures [141,142]. Some researchers suggest that MC3R [143] plays an important role, while others point to the main role of MC4R [144]. The role of MCRs in the anti-inflammatory effects of these peptides has been demonstrated in a number of experiments in vivo and in vitro. The selective MC4R antagonist HS024 has been shown to block the anti-inflammatory effects of α-MSH, preventing the decrease in iNOS expression and the production of NO in astrocytes [145]. Central administration of α-MSH reduces the induction of *iNOS* and *COX-2* gene expression at the hypothalamic level during endotoxemia. The action of α-MSH on LPS-induced iNOS and COX-2 mRNA levels was not observed in the presence of a selective MC4R antagonist HS024 [123]. Pretreatment with another selective MC4R antagonist (HS014) also blocks the behavioral effects induced by IV IL-1β administration [146].

These data indicate that endogenous and synthetic melanocortins exhibit pronounced anti-inflammatory effects both in the periphery and within the brain. Melanocortins, when administered peripherally, can affect inflammatory processes not only in the periphery, but also locally in the brain. It is assumed that depression is associated not only with systemic low-grade inflammation, but also with chronic stress-induced neuroinflammation [147]. Melanocortins act on the peripheral and central inflammatory processes associated with depression, exerting effects similar to those of classical antidepressants. The anti-inflammatory effects of melanocortins are summarized in Table 3.

## 5. The Neuroendocrine Hypothesis of Depression and Melanocortins

The HPAA is one of the most important neuroendocrine systems, and its dysregulation, according to the neuroendocrine hypothesis, underlies the development of depression [148,149]. The functioning of the HPAA and its regulation are complex. Corticotropin-releasing hormone (CRH) (produced predominantly by neurons in the parvocellular division of the paraventricular nucleus (PVN) of the hypothalamus), secreted by the nerve endings of the median eminence, synergistically with arginine vasopressin (AVP) promotes the release of ACTH from the anterior pituitary gland. ACTH stimulates the synthesis and secretion of cortisol by the adrenal cortex. Cortisol inhibits the release of CRH and ACTH by a negative feedback mechanism [150].

The HPAA activity is under the control of various brain structures. An important role belongs to the structures of the limbic system (hippocampus, medial prefrontal cortex, and amygdala) [151]. The hippocampus regulates the HPAA by inhibiting its activity [152,153,154,155]. HPAA activity is also regulated by the medial prefrontal cortex. Lesions of the medial prefrontal cortex significantly increase plasma levels of both ACTH and corticosterone in response to a restraint stress. Injection of corticosterone into the same region of the medial prefrontal cortex produces a significant decrease in plasma levels of both ACTH and corticosterone [156]. The amygdala, on the contrary, stimulates the activity of HPAA. In rats with medial or central amygdaloid nuclei lesions, ACTH and corticosterone responses to stress were blocked [157]. 

Increased levels of cortisol [158] and ACTH [159] indicate dysregulation of HPAA in MDD patients. Such patients have elevated levels of cortisol not only in the blood, but also in saliva [160,161]. Mean adrenal [158] and pituitary [162,163] volumes in depressed patients are significantly larger than the adrenal and pituitary volumes of their matched controls, and this indicates chronic hyperactivation of the HPAA in depression.

In animal models, chronic administration of corticosterone induces depression-like behavior [164,165] and is used as a pharmacological model of depression [166,167]. However, not all researchers confirm the dysregulation of the HPAA in depression [168], because, in some cases, depressed and non-depressed individuals exhibit similar baseline cortisol levels [169,170]. The hypothalamic overdrive and impaired feedback theories of hypercortisolemia in depression are questionable [171]. Such differences in the data may be explained by the fact that not all MDD patients are characterized by abnormalities in the HPAA. It has been shown that the number of depressed patients with disturbances in the HPAA is no more than 27–35% [172]. Normal cortisol levels do not indicate the absence of disturbances. Depressed patients may have disturbed cortisol rhythms [173], or elevated production and clearance rates of cortisol, but a normal 24 h mean plasma cortisol concentration [174]. The relationship between cortisol levels and depression in humans is complex and depends on the disease stage and its severity. Elevated cortisol levels is associated with severe forms of MDD [175].

The dysregulation of HPAA in depression may be associated with impaired functioning of glucocorticoid receptors (GR), through which the regulation of HPAA activity is carried out by a negative feedback mechanism. Malfunction of these receptors may be the cause of HPAA hyperactivation (due to insufficient negative feedback inhibition of HPAA by glucocorticoids) observed in a significant proportion of MDD patients [176]. Glucocorticoid resistance may be a result of impaired glucocorticoid receptors function secondary to chronic exposure to inflammatory cytokines [177]. The dexamethasone suppression test and combined dexamethasone (DEX)/CRH test are considered to measure glucocorticoid receptor-mediated negative feedback and often used as a surrogate marker in depression [178,179], which corresponds to the high clinical significance of the neuroendocrine hypothesis of depression. A meta-analysis confirmed the promise of using the combined DEX/CRH test as a potential diagnostic test for MDD [180].

Successful antidepressant therapy leads to the normalization of the HPAA functioning [181,182,183]. Antidepressants act by restoring the negative feedback regulation of the HPAA using glucocorticoids [176].

However, the question of a causal relationship between effective antidepressant therapy and normalization of HPAA activity is obvious. Does normalization of the HPAA lead to improvement in depressive symptoms, or does the improvement in symptoms normalize the HPAA? If the deregulation of the HPAA causes depression, depressed patients with impaired HPAA could benefit from normalization of its activity. Currently, the possibility of using drugs that reduce the activity of HPAA (antagonists of CRH receptors and glucocorticoid receptors, inhibitors of glucocorticoid synthesis) for the treatment of depression is being investigated [184,185]. However, the therapeutic efficacy of cortisol synthesis inhibitors and glucocorticoid receptor antagonists in depressed patients has not yet been proven. For example, mifepristone, a glucocorticoid receptor antagonist, has been shown to be ineffective [186], and corticosteroid synthesis inhibitors may only be effective in some patients [187]. Patients responding to therapy with cortisol synthesis inhibitors (metyrapone, ketoconazole) tend to have elevated cortisol levels prior to treatment [188]. Clinical trials of CRH1 receptor antagonists have also not yet led to their successful clinical use [184].

Thus, the deregulation of the HPAA is closely associated with depression, and vice versa, the therapeutic effect of antidepressants is associated with the normalization of the HPAA activity. Despite the fact that attempts to develop antidepressant drugs that regulate HPAA activity have not yet led to their clinical use, in our opinion, the possibilities of normalizing HPAA activity are not limited to the effect on CRH receptors and glucocorticoid receptors. The data presented belowindicate the potential for normalizing HPAA activity by targeting the melanocortin system.

ACTH is an activator of all five known melanocortin receptor subtypes and a key player in the HPAA. The production of ACTH by the anterior pituitary gland is under the control of CRH secreted by hypothalamic neurons into the bloodstream, which connects the hypothalamus and pituitary gland. There are several levels of regulation of CRH secretion. CRH secretion in hypothalamic explants is inhibited by dexamethasone, ACTH/α-MSH, and CRH, indicating the existence of three negative feedback loops: an ultrashort CRH-mediated loop, a short loop mediated by hypothalamic POMC peptides (ACTH/α-MSH), and a long glucocorticoid-mediated feedback loop [189]. Permanent ACTH implants into the median eminence significantly depress blood corticosterone levels in rats, which confirms the existence of a short ACTH-mediated negative feedback loop [190]. In adrenalectomized/hypophysectomized animals, subchronic peripheral administration of ACTH significantly reduced the number of CRH- and AVP-positive neurons in the parvocellular division [191] and CRH levels in the hypothalamus [192], which indicates the existence of a short negative feedback loop mediated by circulating ACTH. In rat hypothalamic explants, CRH release is inhibited by ACTH1-39, ACTH1-24, ACTH1-17, and non-corticotropic α-MSH but not by ACTH18-39, further confirming the existence of a short negative feedback loop [193]. Central administration of α-MSH results in a decrease in plasma ACTH levels as well as CRH levels in the median eminence in adrenalectomized rats, indicating the existence of a short negative feedback loop mediated by hypothalamic α-MSH (a loop between CRH in the paraventricular nucleus and peptides derived from POMC in the arcuate nucleus) [194]. MC3R is the most likely candidate of the melanocortin receptors to mediate the short-loop negative feedback release of CRH caused by ACTH/α-MSH peptides [195].

The effects of melanocortins on HPAA activity are poorly understood and seem to depend both on the peptide itself and on the route of administration. Most of the existing studies indicate that central administration of melanocortins lead to activation of the HPAA, and MC4R plays an important role in mediating this effect. Central administration of melanocortin agonist MT-II to conscious and freely moving rats induce a rapid induction of *CRH* gene transcription in the PVN, and this effect is accompanied by a rise in plasma corticosterone levels. MT-II-induced increases in plasma corticosterone is attenuated by the selective MC4R antagonist HS014 [196]. In rats, ICV injection of ACTH1-24, ACTH1-16, and (D-Phe^7^)ACTH4-10 elevates plasma corticosterone levels [197]. The non-selective MC3/4R antagonist SHU 9119 and the selective MC4R antagonist [D-Arg^8^]ACTH4-10, coadministered (ICV) with ACTH1-24, inhibit the ACTH1-24-induced activation of the HPAA, while the selective MC3R agonist Lys-γ2-MSH does not induce activation of the HPAA, which also indicates the important role of MC4R in the activation of the HPAA [198]. Central SHU 9119 administration attenuates the CRH-induced plasma ACTH response [199]. Rats with MC4R loss-of-function have normal basal levels of ACTH and corticosterone. However, the plasma ACTH and corticosterone responses to restraint were significantly reduced by loss of MC4R function. These results support the hypothesis that endogenous MC4R signaling contributes to the HPAA response to stress [200]. The blockade of brain MC4R with intranasal infusion of the MC4R antagonist HS014 to rats prior to single prolonged stress leads to faster termination of stress responses, which is confirmed by a smaller rise in plasma corticosterone [201]. The effects of peripheral melanocortins on HPAA appear to depend on the activity of this neuroendocrine axis. SC administration of α-MSH causes an increase in the plasma corticosterone levels in unstressed rats [202], and blocks their increase caused by acute stress [70]. The above data indicates the HPAA is regulated by melanocortins via ultrashort- and short-loop negative feedback mechanisms. The effects of melanocortins on HPAA activity are summarized in Table 4.

A number of studies show the ability of melanocortins to regulate HPAA activity during inflammation. Central administration of α-MSH, simultaneously with IL-1β, block the IL-1β-induced elevation of plasma ACTH and corticosterone [203]. Central administration of α-MSH prevents the IL-1α-induced increase in cortisol [204]. Peripherally administered melanocortins are also able to regulate the HPAA activity in LPS or cytokine exposed animals. The endotoxin LPS caused a marked increase in plasma ACTH levels in mice, and IP administered α-MSH block LPS-induced ACTH release [205]. IV α-MSH and NDP-α-MSH administration inhibits the capacity of IL-1β to enhance plasma levels of corticosterone [206]. α-MSH and ACTH1-24 exert a dose-dependent inhibitory effect on IL-6-stimulated CRH released from hypothalami explants [207]. Such effects of melanocortins are probably mediated by MC3R/MC4R. Central administration of α-MSH and γ-MSH results in a significant reduction of the IL-1β-induced plasma corticosterone levels. The administration of SHU 9119 or the more selective MC4R antagonist HS014 blocked the effects of peptides [208]. IV administration of NDP-α-MSH significantly attenuates endotoxin-induced levels of proinflammatory cytokines (TNF-α, IL-1β, IL-6), ACTH and cortisol. Selective MC3R agonist D-Trp8-γ-MSH exerts the same effects [209]. IP α-MSH suppresses the LPS-induced rise in plasma ACTH and corticosterone levels. ICV injection of SHU 9119 has no effect on α-MSH-induced suppression of LPS-stimulated plasma corticosterone levels [120], which indicates the possibility of regulation of HPAA activity by blood-brain interface structures, such as the circumventricular organs and vagus nerve.

Therefore, the HPAA activity, both in inflammation and in its absence, can be regulated by melanocortins, including non-corticotropic ones. When administered centrally, MCRs agonists are activators of HPAA, but prevent its activation during central inflammation. When administered peripherally, melanocortin receptor agonists are able to inhibit HPAA activation induced by stress or systemic inflammation. This opens up the possibility of normalizing depression-associated HPAA hyperactivity with peripheral administration of melanocortins.

## 6. The Neurotrophic Hypothesis of Depression and Melanocortins

Neurotrophic factors play an important role in the development and functioning of both the central and peripheral nervous systems, affecting the survival of neurons and their proliferation, the growth of axons and dendrites, and participating in the processes of neurogenesis and synaptic plasticity [210,211]. Changes in the levels of neurotrophic factors may be associated with the pathophysiology of neurodegenerative and mental diseases, including depression. The main facts supporting the neurotrophic hypothesis of depression are as follows: a decrease in the brain-derived neurotrophic factor (BDNF) level in the brain and blood of depressed patients, an increase in the BDNF level after effective antidepressant therapy, and a decrease in the BDNF level in the brains of experimental animals exhibiting depression-like behavior [212,213,214]. Clinical studies show that serum BDNF levels are significantly lower in patients than in controls [215,216]. Effective antidepressant treatment improves serum BDNF levels [217,218,219]. The absence of an early increase of serum BDNF is a highly specific peripheral marker predictive for treatment failure in patients with MDD [220]. Meta-analysis confirms that BDNF levels are abnormally low in patients suffering from MDD and that the BDNF levels are elevated following a course of antidepressant treatment [221]. A higher BDNF promoter methylation status is significantly associated with a suicidal ideation in depression [222]. Post-mortem analysis indicates a decrease in the BDNF level in the hippocampus [223] and distinct cortical areas [224] of depressed patients, as well as in prefrontal cortex and hippocampus of suicide victims [225]. Increased BDNF expression was found in the hippocampus in subjects treated with antidepressant medications at the time of death, compared with antidepressant-untreated subjects [226].

A decrease in BDNF levels has also been demonstrated in experimental models of depression. Chronic unpredictable mild stress (CUMS) induces anhedonia and leads to a decrease in BDNF levels in the hippocampus [227,228]. Data on the BDNF levels in the hippocampus of animals exposed to CUMS are contradictory. Some authors point to a reduction of hippocampal BDNF expression in young (but not adult) rats exposed to CUMS [229], while others observe decreases of BDNF levels in the hippocampus of adult animals and increases of BDNF levels in young animals [230]. Peripheral administration of LPS, used as an inflammatory model of depression, also results in decreased levels of BDNF in the brain [231], reduced expression of BDNF mRNA [232], and reduced BDNF protein levels in the rat hippocampus [233]. Knockdown of BDNF in the dentate gyrus precipitates behaviors associated with depression [234].

BDNF produces antidepressant-like behavioral effects after ICV administration [235], midbrain infusion [236], and a single bilateral infusion into the dentate gyrus of the hippocampus [237]. Chronic administration of various antidepressants causes an increase in BDNF mRNA expression in the hippocampus [238,239,240] and the rat frontal cortex [241]. Interestingly, recent studies demonstrated that antidepressants not only stimulate BDNF expression, but directly bind to the high-affinity TrkB receptor for BDNF, thereby facilitating synaptic localization of TrkB and its activation by BDNF [242,243].

The BDNF-TrkB system interacts with glucocorticoid receptors. Transgenic mice with glucocorticoid receptor-impaired expression display lower levels of BDNF in the hippocampus [244]. GR+/− mice exhibit downregulation of BDNF protein content in the hippocampus and demonstrate increased helplessness after stress exposure. Overexpression of GR in mice evokes reduced helplessness after stress exposure and increases BDNF level in the hippocampus [245].

Such interactions of glucocorticoids with the BDNF-TrkB system influences the central nervous system [246]. Adrenalectomy induces an increase in the hippocampal BDNF mRNA levels, while corticosterone administration decreases BDNF mRNA levels. This may indicate that the *BDNF* gene is under the control of tonic inhibition by glucocorticoids. An excess of glucocorticoids, by repressing the *BDNF* gene, can induce the development of mental disorders [247,248,249]. The interaction of the BDNF-TrkB system with glucocorticoids was also demonstrated in vitro in rat cortical neurons. Cotreatment of dexamethasone and BDNF leads to a change in the expression of 933 genes after 3 h stimulation. Nearly half (455 of 933 genes) of all genes in the BDNF and dexamethasone cohort were uniquely induced or repressed more than 2-fold by cotreatment relative to dexamethasone or BDNF alone. This indicates that a distinct glucocorticoid-responsive transcriptome is evoked upon BDNF signaling. BDNF treatment induces the phosphorylation of GR at serine 155 and serine 287, thereby affecting its transcriptional activity [250]. Glucocorticoids can also affect the BDNF-TrkB system. Glucocorticoids can selectively activate TrkB after in vivo administration in the brain and in cultures of hippocampal and cortical neurons. The activation of TrkB by glucocorticoids does not depend on increased production of neurotrophins. The ability of glucocorticoids to increase TrkB activity results in increased neuronal survival [251].

**Figure 2:**
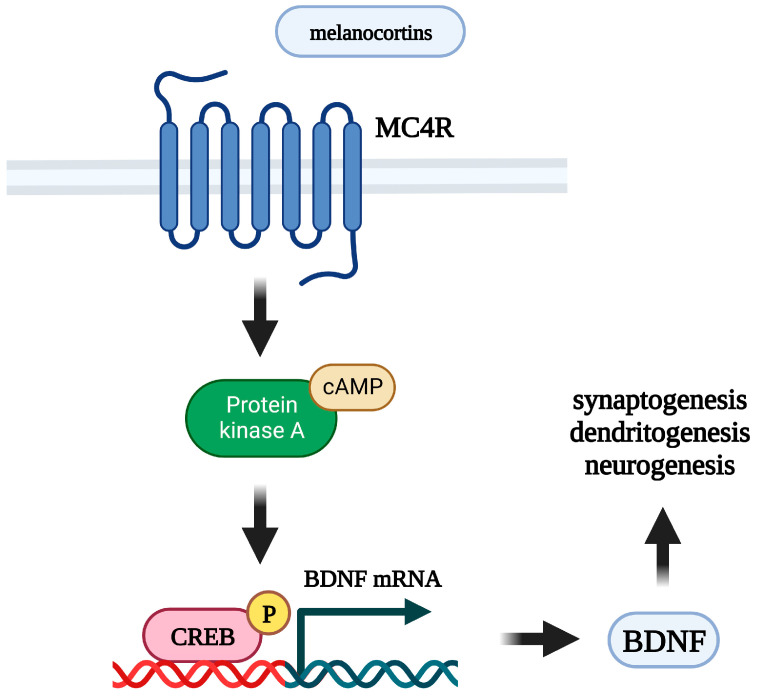
Mechanism of neurotrophic effects of melanocortins. MC4R: melanocortin 4 receptor; BDNF: brain-derived neurotrophic factor; cAMP: cyclic adenosine monophosphate; CREB: cAMP response element-binding protein.

Melanocortins are able to stimulate the levels of neurotrophic factors in the brain in vivo and in cell cultures, mainly by astrocytes (Figure 2). Intranasal administration of Semax results in an increase of BDNF and NGF mRNA transcription [252,253], BDNF protein levels, and expression and activation levels of TrkB receptor [252] in the rat hippocampus. A number of studies point to the relationship between BDNF and the melanocortin system in the context of the control of feeding behavior. In vivo, peripheral administration of MK1 (a selective MC4R agonist) decreases food intake in rats, and this effect is blocked by pretreatment with an anti-BDNF antibody administered into the third ventricle. In vitro, this agonist stimulates BDNF release from isolated rat hypothalami, and this effect is blocked by preincubation with the MC3/4R antagonist SHU 9119 [254]. MC4R^−/−^ mice have a markedly reduced BDNF mRNA level in the ventromedial hypothalamus [255]. However, circulating BDNF concentrations are not significantly associated with MC4R functional status in humans [256].

In addition to the hippocampus and hypothalamus, melanocortins are able to regulate the BDNF level in other brain structures. ICV delivery of the MT-II increases BDNF protein content within the dorsal vagal complex [257]. Acute IP administration of α-MSH increases the levels of BDNF mRNA in the rat striatum [258]. Acute intranasal administration of Semax stimulates BDNF protein levels in the rat basal forebrain [259].

Astrocytes play a key role in the neurotrophic support of neurons, are strongly associated with depression, and are a target for antidepressants [260,261]. In cultured rat brain astrocytes [262,263], the melanocortin receptor agonist NDP-α-MSH induces BDNF mRNA transcription. In rat basal forebrain astrocytes, Semax stimulates BDNF and NGF mRNA levels [264]. In cultured rat cortical astrocytes, α-MSH increases expression of vascular endothelial growth factor (VEGF) [265], which is a potent neurotrophic factor and is required for neurotrophic and antidepressant effects of BDNF [266]. In addition to the effects of melanocortins on astrocytes, resent data shows that NDP-α-MSH stimulates BDNF mRNA expression in murine Neuro2a neuroblastoma cells [267].

Thus, melanocortin agonists demonstrate an ability similar to antidepressants to stimulate the expression of neurotrophic factors in the hippocampus, a number of other brain regions, and in cultured astrocytes. The neurotrophic effects of melanocortins are summarized in Table 5.

## 7. The Neurogenesis Hypothesis of Depression and Melanocortins

The pathophysiology of depression is also associated with impaired synaptic plasticity and such processes as neurogenesis, synaptogenesis, dendritogenesis, and axon branching. New neurons in adult mammals are formed only in two areas of the brain: the subventricular zone of the lateral ventricles, and the subgranular zone of the dentate gyrus of the hippocampus. The depression hypothesis associated with impaired neurogenesis postulates that a decrease in the formation of new neurons in the dentate gyrus is associated with the pathophysiology of depression, and an increase in neurogenesis in the hippocampus is necessary for antidepressants to exert their therapeutic effects [268]. The results of several meta-analyses indicate a decrease in hippocampal volumes in depressed patients by an average of 8–10% compared with healthy people [269,270,271,272,273]. In addition to the hippocampus, a decrease in volume is also observed in frontal regions, the putamen, and the caudate nucleus [274]. It is not clear whether the decrease in hippocampal volume is associated with inhibition of neurogenesis, but impaired neurogenesis may be one of the causes of the observed atrophy. Reduced hippocampal neurogenesis is not a cause of stress-related behavioral deficits in animals [275], but behavioral effects of chronic antidepressants treatment may be mediated by the stimulation of neurogenesis in the hippocampus [276]. Chronic antidepressant treatment induces neurogenesis in adult rat hippocampus increasing the proliferation of cells in the dentate gyrus [277].

The hippocampus is believed to be highly sensitive to stress, and high levels of glucocorticoids may induce hippocampal atrophy [278]. Dentate granule cells are enriched with mineralocorticoid and glucocorticoid receptors. These neurons require hormone levels to be within the physiological range. In the absence of corticosteroids, proliferation and apoptotic cell death are dramatically enhanced. Dendritic morphology and synaptic transmission are compromised. Prolonged high levels of corticosteroids conversely suppress neurogenesis [279]. The role of neurogenesis on the development of depressive symptoms is largely unexplored. Neurogenesis in the hippocampus may be necessary to maintain normal HPAA activity through a hippocampus-dependent negative feedback mechanism. If it is true, then patients with HPAA hyperactivation will be characterized by reduced neurogenesis in the hippocampus, and stimulation of neurogenesis will normalize HPAA activity.

The ability of melanocortins to stimulate brain BDNF expression indicates the possibility of enhancing of hippocampal neurogenesis, since the BDNF-TrkB system is deeply involved in this process [280]. NDP-α-MSH stimulates neurogenesis, which increased the proliferation of neural progenitors in the hippocampus in an experimental model of cerebral ischemia [281,282] and in a model of Alzheimer’s disease [283]. Subchronic IP administration of selective MC4R agonist RO27-3225 enhances neurogenesis in the subventricular zone in a model of cerebral infarction [284]. These data show that under pathological conditions, melanocortins induce neurogenesis both in the subventricular zone of the lateral ventricles, and in the subgranular zone of the dentate gyrus. It should be noted that in all the above cases, the authors point to the involvement of MC4R in the observed effects. Thus, the available data suggest that melanocortins can enhance adult hippocampal neurogenesis, although more research is needed in this area. If the effects of melanocortin agonists are confirmed in further studies using experimental models of depression, it could be hypothesized that melanocortins may be effective in normalizing depression-related impairments of hippocampal neurogenesis.

## 8. The Glutamate Hypothesis of Depression and Melanocortins

The glutamate hypothesis of depression was proposed following the discovery of the antidepressant-like effects of NMDA glutamate receptor (NDMAR) antagonists in mice [285]. In clinical trials, antidepressant effects were confirmed for the NDMAR antagonist ketamine [31], but not for a number of other NMDAR antagonists, such as memantine and MK-801. The glutamate hypothesis proposes that depression is associated with altered glutamatergic excitation at synapses in limbic regions [286]. Although the precise mechanism of ketamine’s antidepressant action is still unclear, according to the glutamate hypothesis, a simplified mechanism involves blocking of NMDAR by ketamine, which causes the enhanced release of glutamate into the synaptic cleft. This leads to increased activation of AMPA glutamate receptors (AMPAR) and a subsequent increase in expression and release of BDNF, which normalizes impaired synaptic transmission [287].

In this context, it is interesting that ACTH and a number of its corticotropic fragments inhibit binding of the selective NMDAR ligand MK-801 to hippocampal membranes in vitro [288]. α-MSH has also been shown to reduce the activation of NMDAR and non-NMDAR (AMPAR and/or metabotropic group I glutamate receptors) in rat striatal slices [289]. In ex vivo binding studies in mice, peripheral administration of Semax has been shown to decrease the density of hippocampal NMDAR [290].

With regard to AMPAR, Semax was found to potentiate AMPAR currents in rat cerebellum Purkinje cells in vitro [291]. An in vitro study also showed that activation of postsynaptic MC4Rs in rat hippocampal neurons increased surface expression of the GluA1 subunit of the AMPAR and enhanced synaptic transmission [292]. In the same study, IP injection of the melanocortin agonist d-Tyr MT-II or MT-II was shown to enhance long-term potentiation (LTP) in the CA1 region of the mouse hippocampus. However, activation of MC4Rs in the NAc by α-MSH results in suppression of synaptic transmission mediated by AMPAR [293], indicating region-specific effects of melanocortins. Quite remarkably, a similar situation has been demonstrated for ketamine, which enhances AMPAR function in the hippocampus and medial prefrontal cortex but impairs it in the NAc [294].

Thus, despite the limited amount of available data, central and peripheral melanocortin agonists are able to affect both NMDAR and AMPAR function, and exhibit effects consistent with the mechanism of antidepressant action proposed by the glutamate hypothesis of depression.

## 9. The Endocannabinoid Hypothesis of Depression and Melanocortins

According to the endocannabinoid hypothesis of depression, changes in the levels of endogenous cannabinoids and/or the functioning of their receptors in the brain cause symptoms of depression [295]. Depressed patients show a decrease in circulating endocannabinoids levels, and in turn, successful antidepressant treatment increases their levels. Blockade of the most abundant endocannabinoid receptor in the brain, CBR1, causes depression, but chronic activation of CBR1 induces their desensitization. At the same time, depression is associated with an increased density of CBR1 in the prefrontal cortex, which is assumed as a compensatory mechanism in response to reduced levels of endocannabinoids [295,296]. Both the melanocortin and endocannabinoid systems in the hypothalamus are involved in the regulation of eating behavior, energy homeostasis [297], and HPAA activity [298], suggesting their close interactions. However, ICV administration of α-MSH did not affect hypothalamic levels of endocannabinoids, but administration of HS014, a selective MC4R antagonist, increased their levels [299]. However, it is not yet known whether central or peripheral melanocortins affect endocannabinoid levels and their receptors in brain regions associated with depression, such as the limbic system and prefrontal cortex.

## 10. The Effect of Melanocortins on Depression-like and Anxious Behavior

The question of the endogenous level of melanocortins in depression remains open due to the small number of studies on this topic. Some researchers indicate a reduced plasma level of α-MSH in MDD patients [300], other authors do not detect any differences in the plasma level of α-MSH between MDD patients and healthy controls [301]. There were no differences between depressed patients and healthy controls in α-MSH and ACTH levels in cerebrospinal fluid and plasma [302]. There are currently no data on the effect of melanocortins on depressive and anxious behavior in humans. The only exception is a study, which showed that IV administration of an ACTH/MSH4-10 to human subjects leads to a decrease in anxiety [303].

Sequence polymorphisms of MCR genes may contribute to the risk of major depressive disorder. It has been shown that the rs885479 polymorphism in the *MC1R* gene [304], rs111734014 polymorphism in the *MC2R* gene, and rs2236700 in the *MC5R* gene are associated with the risk of MDD [305].

Indirectly, the possible involvement of the melanocortin system in depression is indicated by studies demonstrating a close relationship between depression and obesity [306,307]. A change in appetite (and consequently a change in body weight) is one of the symptoms of depression. In turn, melanocortins are important regulators of feeding behavior [308,309,310,311].

The role of melanocortins in animal models of anxiety and depression is being actively studied. Central endogenous α-MSH may be involved in the development of anxiety and depression. Most studies point to the antidepressant and anxiolytic properties of melanocortin receptor antagonists and the anxiogenic effects of agonists. Antidepressant and anxiolytic properties are exerted by central and peripheral administration of selective MC4R antagonists, such as HS014 [312], MCL0129 [313], MCL0042 [314], and the MC3R/MC4R antagonist SHU 9119 [315,316]. Intranasal infusion of HS014 also prevents development of depressive-like and anxiety-like behavior [317,318]. The important role of MC4R allows for it to be considered as a target for the development of drugs for the treatment of stress-associated diseases, such as anxiety and depression [319]. Centrally administered MCRs agonists exert anxiogenic effects. The level of anxiety increases after central administration of α-MSH [320,321] and ACTH1-24 [322], but not of ACTH4-10 and ACTH11-24. Similar effects have been observed after α-MSH administration into the medial preoptic area [323]. MC4R signaling in the dorsal raphe nucleus affects anxiety and depression-like behavior [324]. However, centrally administered melanocortins may exert quite the opposite effects antagonizing the action of cytokines. Central administration of α-MSH reverses IL-1β-induced anxiety and administration of HS014 inhibit the effect of α-MSH [325].

The effects of melanocortin receptor agonists on depression-like behavior are even more controversial. Some authors point to the prodepressant properties of α-MSH after central administration [326], while others do not demonstrate any influence of α-MSH on the depression-like behavior [327]. Peripherally administered melanocortins exert antidepressant effects. IP administration of α-MSH (but not ACTH4-10 and ACTH1-24) decrease immobility in the forced swim test [328]. IP administered ACTH6-9-Pro-Gly-Pro also exerts antidepressant and anxiolytic effects [329]. We have previously shown that peripheral administration of α-MSH and ACTH4-10 attenuates anhedonia in an inflammatory and CUMS models of depression [330].

Several studies have shown that chronic administration of ACTH blocks the effects of antidepressants. A single administration of either imipramine or desipramine significantly decreases the duration of immobility in normal rats. The immobility-decreasing effect induced by a single administration of antidepressants is blocked by chronic administration of ACTH1-24, which like a full-sized ACTH, possesses corticotropic activity [331,332,333].

Antidepressants can also affect the melanocortin system. Fluoxetine administration increases POMC expression and reduces MC4R expression in the hypothalamus [334]. POMC mRNA levels in the arcuate nucleus of the hypothalamus are increased following chronic treatment with phenelzine and idazoxan [335]. However, orally administered fluoxetine decrease α-MSH levels in the PVN of the hypothalamus [336].

The above data indicate the involvement of the melanocortin system in the development of depressive-like and anxious behavior. The inconsistency of these data indicates the need for further research in this area. The effects of agonists and antagonists of melanocortin receptors depend on the route of administration (central or peripheral), the ability of drugs to cross the blood-brain barrier, the specific area into which the drug is administered when it is administered centrally, and the dose and selectivity of the agonist/antagonist to MCRs. The effects of melanocortins on depression-like and anxious behavior are summarized in Table 6.

## 11. The Role of Melanocortins in Motivational and Hedonic Behavior

Melanocortins are involved in the regulation of feeding behavior. Central administration of melanocortin receptor agonists decrease food intake. Melanocortins are able to regulate not only homeostatic (metabolic), but also motivational and hedonic aspects of feeding behavior, which is of particular interest from the point of view of anhedonia. Anhedonia is most often assessed by the sucrose preference test in experimental models.

MC4R deficient individuals exhibit a significantly reduced preference for high sucrose food [337]. Deletion of both alleles of the MC4R decreases preference for palatable high-sucrose foods in wild-type mice [338]. Global deletion of the MC3R decreases sucrose intake and preference in female but not male mice [339].

The importance of the melanocortin system in the regulation of the motivational and consummatory phases of food consumption is evidenced by animal studies using melanocortin receptor agonists and antagonists. MT-II injected into the NAc decrease both appetitive (motivational) and consummatory feeding behavior in mice [340]. Chronic stress-elicited anhedonia requires activation of MC4R in the NAc [293]. Injection of MT-II into the VTA decreases motivation to obtain sucrose pellets on both fixed ratio and progressive ratio schedules of reinforcement [341] and decreases the intake of sucrose solution [342]. Intra-VTA infusion of the selective MC3R agonist γ-MSH, on the contrary, increases responding for sucrose under a progressive ratio schedule of reinforcement [343]. MC4R in the dorsomedial striatum appears to propel reward-seeking behavior [344].

Food motivated behavior tested under a progressive ratio schedule of reinforcement dose-dependently decreased by ICV-injected α-MSH. In contrast to progressive ratio responding, free intake of sucrose remains unaltered upon α-MSH infusion. The authors suggest that the motivation for palatable food is modulated by MC4R in the NAc [345]. Central AGRP administration results in significantly increased motivation for sucrose solution in rats under a progressive ratio schedule of reinforcement [346]. Chronic central MCR ligand infusion (SHU 9119 and MT-II) does not affect the response to non-ingestive reward stimuli (lateral hypothalamic electrical stimulation) [347]. However, ICV infusion of α-MSH decreases the rewarding properties of social interactions (rewarding stimulus) in Syrian hamsters [348].

The effect of melanocortins on the perception of aversive stimuli was also shown. In normal mice, systemic inflammation induced by LPS administration, results in aversion in a conditioned place aversion paradigm. In contrast, mice lacking MC4R display preference toward the aversive stimuli. Intranasal administration of MC4R antagonist HS014 prior to LPS injection to wild-type mice results in antiaversive effect. This means that MC4R signaling is required for assigning negative motivational valence to aversive stimuli [349].

The mechanisms of the regulatory effects of melanocortins on the motivational and hedonic aspects of feeding behavior are currently unknown. The interaction of the melanocortin and dopaminergic systems can play an important role. Hyperactivity of POMC neurons in the arcuate nucleus of the hypothalamus (POMC^ARH^ neurons) results in decreased neural activities of dopamine neurons in the VTA. Inhibition of the POMC^ARH^→VTA circuit reduces depression-like behavior and anhedonia in mice exposed to chronic restraint stress [350]. α-MSH infusion into the lateral hypothalamic area decreases food intake and sucrose consumption and increases dopamine levels in rats. Dopamine release occurs in both the anticipatory and consummatory phases of feeding. These data suggest that α-MSH-stimulated activation of the dopaminergic system is involved in homeostatic and hedonic satiation [351].

These data indicate the involvement of central melanocortin receptors in the regulatory mechanisms of the motivational and hedonic aspects of feeding behavior, and the close relationship between the melanocortin and dopaminergic systems. Virtually all studies point to the ability of melanocortin receptor agonists, after their central administration, to suppress motivation for food rewards and reduce the consumption/preference for palatable food. However, nothing is known about the effects of melanocortins after their peripheral administration in this context.

## 12. Some Features of Melanocortins and Their Possible Site of Action

Melanocortins are often injected ICV or directly into those brain structures that are of interest in a particular study. The central route of administration is unacceptable for humans and preference is given to peripheral routes of administration. But the peripheral route of administration for peptides also has limitations due to their rapid degradation by peptidases. The half-life of α-MSH in plasma is about 7–18 min and depends on the acetylation status of the peptide [352]. However, peptides have a number of important advantages, including high affinity, specificity for receptors, as well as low immunogenicity and toxicity. There are approaches that improve the absorption properties of peptides, increase their proteolytic stability, and reduce renal clearance. Among the strategies that are often used in the creation of drugs based on peptides are: molecule cyclization, N-terminus acetylation, replacement of L-amino acids with D-amino acids, the use of non-canonical amino acids, and conjugation to other molecules [353,354].

The nature of the effects after the peripheral administration of melanocortins indicates their central action, but α-MSH does not cross the blood-brain barrier [355]. How melanocortins exert central effects after peripheral administration remains unknown. Circumventricular organs may play an important role in this process. Melanocortins [356] and their binding sites [357,358,359] were found in the median eminence. Probably, different members of the melanocortin family differ in their ability to cross the blood-brain barrier. A synthetic analogue of melanocortins (Semax) penetrates into the brain both after IV [360] and after intranasal administration [361]. In contrast, MT-II, a synthetic analog of α-MSH, does not cross the blood-brain barrier after IV administration and is detectable only in circumventricular organs [362]. It is possible that small-molecules agonists and antagonists of melanocortin receptors will be able to cross the blood-brain barrier much more easily.

## 13. Conclusions

Despite poorly understood molecular and cellular mechanisms of depression, accumulated data indicate that the pathophysiology of this disease is associated with disturbances in closely interrelated nervous, immune, and neuroendocrine systems. These disruptions affect patterns of synaptic activity in the brain, which is reflected in changes in consciousness, behavior, mood, and emotions [363]. The complexity of the mechanisms that cause depression-associated states of the nervous system corresponds to the existence of several hypotheses of the causes of depression and the mechanisms of action of antidepressant drugs. Insufficient effectiveness of available antidepressants requires the search for new antidepressant medications. One strategy for such a search could be to consider the known activities of a candidate compound or family of compounds in the light of existing hypotheses of depression. 

**Figure 3:**
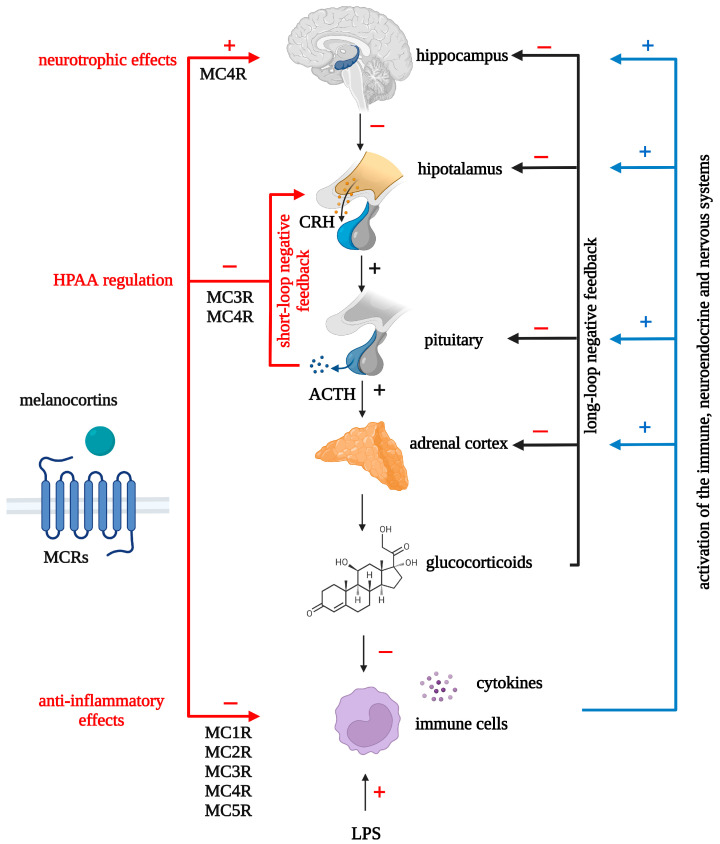
Neurotrophic, neuroendocrine and anti-inflammatory effects of melanocortins. MCRs: melanocortin receptors; MC1R: melanocortin 1 receptor; MC2R: melanocortin 2 receptor; MC3R: melanocortin 3 receptor; MC4R: melanocortin 4 receptor; MC5R: melanocortin 5 receptor; ACTH: adrenocorticotropic hormone; CRH: corticotropin-releasing hormone.

The melanocortin system is involved in the regulation of numerous biological processes, such as body weight and energy balance control, behavior, cognition, and neuroprotection. The most investigated melanocortins, ACTH and α-MSH, are key players in the body’s neuroendocrine response to stress. An analysis of the known activities of melanocortins shows that they are able to influence those body’s systems that are involved in depression and which are normalized by effective antidepressant therapy. Melanocortins, including non-corticotropic fragments, exhibit anti-inflammatory effects, are able to affect brain levels of neurotrophic factors, and stimulate neurogenesis in the hippocampus in some pathological states (Figure 3). There is evidence that non-corticotropic melanocortins can affect brain monoamine levels, enhance AMPAR-mediated hippocampal synaptic transmission, and inhibit HPAA activity via glucocorticoid-independent negative feedback. An important feature of melanocortins is their ability to have central effects when administered peripherally. There are some open questions regarding potential treatment with melanocortins. Do melanocortins need to cross the blood-brain barrier for their central and neuroendocrine effects? What receptor subtypes are responsible for certain effects of melanocortins and where are these receptors located? What negative side effects can be expected if they are used to treat depression? To formulate research and development strategies for potential melanocortin-based antidepressants, we need to answer these questions.

The polyfunctionality of melanocortins and their potential safety may be an important advantage of these peptides over other compounds with antidepressant properties. Simultaneous normalization of pro-inflammatory cytokines, cortisol, and neurotrophic factors, as well as stimulation of neurogenesis induced by melanocortin treatment, may result in faster and more effective improvement in depressive symptoms. The availability of melanocortin-based drugs already approved for clinical use greatly facilitates the possibility of testing them in depressed patients. The effectiveness of peripherally and, in particular, intranasally administered synthetic melanocortins analogues is an important advantage of these peptides that creates the basis for the synthesis of new, more effective, melanocortin-based molecules.

Given that depression is associated with disturbances in a number of physiological processes, and its pathogenesis may include various paths, the creation of a single universal remedy for the treatment of this affective disorder is hardly possible. However, melanocortins could be used in the complex therapy of depression along with other treatments. To establish this possibility, additional fundamental and applied studies of the central and peripheral effects of endogenous and synthetic melanocortins and their mechanisms of action are needed.

## Figures and Tables

**Table 1:** Amino acid sequences of natural melanocortins.

Peptide	Amino Acid Sequence
ACTH	Ser-Tyr-Ser-Met-Glu-His-Phe-Arg-Trp-Gly-Lys-Pro-Val-Gly-Lys-Lys-Arg-Arg-Pro-Val-Lys-Val-Tyr-Pro-Asn-Gly-Ala-Glu-Asp-Glu-Ser-Ala-Glu-Ala-Phe-Pro-Leu-Glu-Phe
α-MSH	Ac-Ser-Tyr-Ser-Met-Glu-His-Phe-Arg-Trp-Gly-Lys-Pro-Val-NH_2_
β-MSH	H-Ala-Glu-Lys-Lys-Asp-Glu-Gly-Pro-Tyr-Arg-Met-Glu-His-Phe-Arg-Trp-Gly-Ser-Pro-Pro-Lys-Asp-OH
γ1-MSH	H-Tyr-Val-Met-Gly-His-Phe-Arg-Trp-Asp-Arg-Phe-NH2
γ2-MSH	H-Tyr-Val-Met-Gly-His-Phe-Arg-Trp-Asp-Arg-Phe-Gly-OH
γ3-MSH	H-Tyr-Val-Met-Gly-His-Phe-Arg-Trp-Asp-Arg-Phe-Gly-Arg-Arg-Asn-Ser-Ser-Ser-Ser-Gly-Ser-Ser-Gly-Ala-Gly-Gln-OH

**Table 2:** A summary of the effects of melanocortin agonists on dopaminergic, serotonergic and noradrenergic systems.

Subjects	Experimental Model	Agonist	Dose	Site of Injection	Outcome	Agonist Effects Inhibitor	Dose	Site of Injection	Ref.
Studies performed in vivo									
Male albino rats	Conscious and freely moving rats	α-MSH	1 μg	VTA	Dopamine ↓ and DOPAC ↑ in the caudate putamen and nucleus accumbens	-	-	-	[61]
Long–Evans rats	Conscious and freely moving rats	AgRP (inverse agonist)	1 nmol	ICV	Neuronal activation within midbrain dopamine neurons and dopamine turnover in the medial prefrontal cortex ↑	-	-	-	[62]
Male SD rats	Anaesthetized rats	α-MSH	10 nmol	VTA	Dopamine and DOPAC levels in the nucleus accumbens ↑	HS131	1 nmol	VTA	[64]
Male Wistar rats	Conscious and freely moving rats	MT-II	0.0625, 0.625 µg	ICV	Changes in dopamine D1-like and D2-like receptor binding in several brain regions	-	-	-	[65]
Male Wistar rats	Anaesthetized rats	MT-II	3 nmol	ICV	Firing rate of locus coeruleus noradrenergic neurons ↓ and firing rate of dorsal raphe nucleus serotonergic neurons ↑	-	-	-	[67]
		AgRP (inverse agonist)	1 nmol	ICV	Firing rate of locus coeruleus noradrenergic neurons ↑				
Male C57BL/10 Bg mice	Conscious and freely moving mice	ACTH	20 IU/kg	IP	Tyrosine hydroxylase activity in the locus coeruleus ↑	-	-	-	[68]
		ACTH1-24	20 μg/kg						
		ACTH4-10	20 μg/kg						
Male SD and Wistar rats	Conscious and freely moving mice	ACTH4-10	25, 50 µg	SC	Catecholamine synthesis in the whole brain and brain stem ↑	-	-	-	[69]
Male C57/bl mice and male SD rats	Conscious and freely moving rats and mice	Semax	0.15 mg/kg	IP	Extracellular striatal level of 5-HIAA ↑	-	-	-	[71]
Studies performed in vitro									
Male Wistar rats	Striatal tissue	ACTH1-24	10 μM	-	Inhibition of association and dissociation of dopamine D2 agonist to the dopamine D2 receptor	-	-	-	[66]
Male SD rats (synaptosomal preparation from cortical areas)	FST	α-MSH	1, 4 µg	SC	Inhibition of FST-activated [^3^H]-5-HT re-uptake	-	-	-	[70]

↓: decrease; ↑: increase; SD: Sprague–Dawley rats; ICV: intracerebroventricular; SC: subcutaneous; IP: intraperitoneal; FST: Porsolt’s forced swim test; VTA: ventral tegmental area.

**Table 3:** A summary of the effects of endogenous and synthetic melanocortin agonists in different models of inflammation.

Subjects	Experimental Model	Agonist	Dose	Site of Injection	Outcome	Agonist Effects Inhibitor	Dose	Site of Injection	Ref.
Studies performed in vivo									
White rabbits	LPS-induced fever	α-MSH	2.5 µg	IV	Antipyretic effect	-	-	-	[116]
			200 ng	ICV					
White rabbits	IL-6 and TNF-α-induced fever	α-MSH	200 ng	ICV	Antipyretic effect	-	-	-	[117]
White rabbits	Leukocytic pyrogen-induced fever	α-MSH	200 ng	ICV	Antipyretic effect	-	-	-	[118]
		NDP-α-MSH	10, 20 ng						
Male SD rats	LPS-induced fever	α-MSH	300 ng/rat	ICV	Antipyretic effect	SHU 9119	200 ng	ICV	[119]
Male SD rats	LPS-induced fever	α-MSH	25–100 µg/kg	IP	Antipyretic effect; IL-6 ↓	SHU 9119	200 ng	ICV	[120]
Male SD rats	LPS-induced fever	α-MSH	1 µg	ICV	Antipyretic effect	HS014	1 µg	ICV	[121]
Male Wistar rats	LPS-induced endotoxemia	α-MSH	3 nmol/rat	ICV	iNOS, COX-2 ↓	HS024	1 nmol/rat	ICV	[123]
Male BALB/c mice	LPS challenge	α-MSH	10 µg	ICV	TNF-α ↓	-	-	-	[124]
			50 µg	IP					
Male Wistar rats	Transient cerebral ischaemia	Semax	10 µg/100 g	IP	Expression of genes related to inflammatory processes ↓	-	-	-	[125]
Male Wistar rats	Transient focal cerebral ischaemia	Semax	10 µg/100 g	IP	IL-1α, IL-1β, IL-6, Ccl3, Cxcl2 ↓	-	-	-	[126]
Unsexed SD rat pups	Neonatal hypoxia-ischemia brain injury	BMS-470539	50, 160, 500 μg/kg	Intranasal	IL-1β, TNF-α, IL-6 ↓	-	-	-	[127]
Male Swiss Albino mice	Experimental gouty arthritis	ACTH4–10	100 µg	SC	KC, IL-1β ↓	SHU 9119	10 µg	IP	[137]
		α-MSH	10 µg						
		β-MSH	10 µg						
Studies performed in vitro									
Rat hypothalamic explants	LPS + IFN-γ	α-MSH	5 µM	-	iNOS ↓	-	-	-	[123]
Mice brain tissue	LPS challenge	α-MSH	10^−15^–10^−8^ M	-	TNF-α ↓	-	-	-	[124]
Murine microglial cell line (N9)	LPS + IFN-γ	α-MSH	1, 10, 25, 50, 100 µM	-	NO, TNF-α, IL-6 ↓	-	-	-	[128]
		α-MSH11–13							
		ACTH1–24							
Anaplastic astrocytoma cell line A-172	LPS + phorbol 12-myristate 13-acetate	α-MSH	10^−17^–10^−10^ M	-	TNF-α ↓	-	-	-	[129]
Human astrocytoma cells (U373)	TNF-α + IFN-γ	Setmelanotide	0.001–10 µM	-	CCL2, CXCL10 ↓IL-6, IL-11 ↑	SHU 9119	10 µM	-	[130]
RAW 264.7 cells	LPS + IFN-γ	α-MSH	5 × 10^−13^–5 × 10^−5^ M	-	Nitrite accumulation, iNOS ↓	-	-	-	[132]
Human monocyte-derived dendritic cells	-	α-MSH	10^−12^ M	-	CD86, CD40 ↓	-	-	-	[135]
Mice peritoneal macrophages	Monosodium urate crystals	ACTH	ACTH (100 ng/mL)	-	KC, phagocytosis ↓	-	-	-	[137]
		ACTH4–10	100 µg/mL						
		α-MSH	10 µg/mL						
		β-MSH	10–30 µg/mL						
Cultured rat astrocytes	LPS + IFN-γ	α-MSH	5 µM	-	NO, PGE2, iNOS, COX-2 ↓	HS024	0.5 µM	-	[145]

↓: decrease; ↑: increase; SD: Sprague–Dawley rats; ICV: intracerebroventricular; IV: intravenous; SC: subcutaneous; IP: intraperitoneal.

**Table 4:** A summary of the effects of melanocortin agonists on HPAA activity.

Subjects	Experimental Model	Agonist	Dose	Site of Injection	Outcome	Agonist Effects Inhibitor	Dose	Site of Injection	Ref.
Studies performed in vivo									
Male SD rats	ACTH implants into the median eminence	ACTH	5 U	Median eminence	Corticosterone ↓	-	-	-	[190]
Male SD rats	Adrenalectomized and hypophysectomized animals	ACTH	0.5, 2 mg/mL (1 μL/h)	SC	Number of AVP- and CRF-positive PVN neurons ↓	-	-	-	[191]
Male SD rats	Adrenalectomized and hypophysectomized animals	ACTH	2 U	SC	CRH ↓	-	-	-	[192]
Male Wistar rats	Adrenalectomized animals	α-MSH	166, 1663 ng	ICV	Plasma ACTH levels ↓ CRF levels in the median eminence ↓	-	-	-	[194]
Male SD rats	Conscious and freely moving rats	MT-II	0.5, 1 nmol	ICV	Plasma corticosterone and CRH mRNA ↑	HS014	0.25, 0.5, 1.0 nmol	ICV	[196]
Male Wistar rats	Conscious and freely moving rats	[D-Phe^7^]ACTH4-10	5 µg	ICV	Plasma corticosterone ↑	-	-	-	[197]
		ACTH1-16	1 µg						
		ACTH1-24	1 µg						
Male Wistar rats	Conscious and freely moving rats	ACTH1-24	0.3–3 µg	ICV	Plasma ACTH and corticosterone ↑	SHU 9119	1, 3 µg	ICV	[198]
						[D-Arg^8^]ACTH4-10	5, 10 µg	ICV	
Male Wistar rats	Acute central CRH administration	-	-	-	Plasma ACTH levels ↓	SHU 9119 (solo effect)	0.5 nmol/day over 14 days	ICV	[199]
Male rats	Homozygous or heterozygous male rats with loss of function mutation in MC4R	-	-	-	Stress-induced increase in circulating ACTH and corticosterone was reduced by loss of MC4R function	-	-	-	[200]
Male SD rats	Single prolonged stress	-	-	-	Plasma corticosterone ↓	HS014 (solo effect)	100 µg	Intranasal	[201]
Male Wistar rats	Conscious and freely moving rats	α-MSH	50, 500 µg	SC	Plasma corticosterone ↑	-	-	-	[202]
Male SD rats	FST	α-MSH	1, 4 µg	SC	Plasma corticosterone ↓	-	-	-	[70]
Male SD rats	IL-1β infusion	α-MSH	0.1, 1.0, 10 ng	ICV	Plasma ACTH and corticosterone ↓	-	-	-	[203]
Female rhesus monkeys	IL-1α infusion	α-MSH	120 µg/h for 2 h	ICV	Plasma cortisol ↓	-	-	-	[204]
Male BALB/c mice	LPS and IL-1β-induced HPAA activation	α-MSH	10 and 30 µg	IP and SC	Plasma ACTH ↓	-	-	-	[205]
CR:SW mice	IL-1β-induced HPAA activation	α-MSH	30 µg	IV	Plasma corticosterone ↓	-	-	-	[206]
		NDP-α-MSH	15 µg						
Male Wistar rats	IL-1β-induced HPAA activation	γ-MSH	1 µg	ICV	Plasma corticosterone ↓	SHU 9119	1 µg	ICV	[208]
		α-MSH	0.1 µg			HS014	1 µg	ICV	
Female rhesus monkeys	LPS-induced HPAA activation	NDP-α-MSH	20 µg/h for 7 h	IV	Plasma ACTH and cortisol ↓	SHU 9119	20 µg/h for 7 h	IV	[209]
Male SD rats	LPS-induced fever	α-MSH	100 µg/kg	IP	Plasma ACTH and corticosterone ↓	SHU 9119	200 ng	ICV	[120]
Studies performed in vitro									
Rat hypothalamic explants	5HT-, ACh-, and NE-stimulated CRH secretion	ACTH	10^−10^–10^−7^ M	-	CRH ↓	-	-	-	[189]
		α-MSH							
Rat hypothalamic explants	Rat hypothalamic perifusion system	ACTH	0.22–22 nM	-	CRH ↓	-	-	-	[193]
		ACTH1-24	2.2 nM						
		ACTH1-17	2.2 nM						
		α-MSH	2.2 nM						
Rat hypothalamic explants	IL-6-stimulated CRH release	ACTH1-24	10^−15^–10^−13^ M	-	CRH ↓	-	-	-	[207]
		α-MSH	10^−13^–10^−11^ M						

↓: decrease; ↑: increase; SD: Sprague–Dawley rats; ICV: intracerebroventricular; IV: intravenous; SC: subcutaneous; IP: intraperitoneal; FST: Porsolt’s forced swim test.

**Table 5:** A summary of the effects of melanocortin agonists on BDNF mRNA and protein levels.

Subjects	Experimental Model	Agonist	Dose	Site of Injection	Outcome	Agonist Effects Inhibitor	Dose	Site of Injection	Ref.
Studies performed in vivo									
Male rats	Conscious and freely moving rats	Semax	50, 500 μg/kg	Intranasal	Hippocampal BDNF protein levels ↑	-	-	-	[252]
Male Wistar rats	Conscious and freely moving rats	Semax	50 μg/kg	Intranasal	Hippocampal BDNF mRNA levels ↑	-	-	-	[253]
MC4R^−/−^ null mice	MC4R^−/−^ null mice	-	-	-	Hypothalamic BDNF mRNA levels ↓	-	-	-	[255]
Male Wistar Han rats	Conscious and freely moving rats	MT-II	1 nmol/rat	ICV	BDNF protein levels within the dorsal vagal complex ↑	SHU 9119	0.5 nmol/rat	ICV	[257]
Male Wistar rats	Conscious and freely moving rats	α-MSH	0.5 mg/kg	IP	Striatal BDNF mRNA ↑	-	-	-	[258]
Male Wistar rats	Conscious and freely moving rats	Semax	50, 250 μg/kg	Intranasal	BDNF protein levels within the basal forebrain ↑	-	-	-	[259]
Male Wistar rats	Conscious and freely moving rats	α-MSH	0.5 mg/kg	IP	Hypothalamic BDNF mRNA levels ↑	-	-	-	[263]
Studies performed in vitro									
Rat hypothalamic explants	Isolated rat hypothalami	MK1	1, 10 µM	-	BDNF release ↑	SHU 9119	3 µM	-	[254]
Rat astrocytes	Rat cultured astrocytes	NDP-α-MSH	0.1, 1, 10 μM	-	BDNF mRNA and protein levels ↑	-	-	-	[262]
Rat astrocytes	Rat cultured astrocytes	NDP-α-MSH	1 μM	-	BDNF mRNA ↑	-	-	-	[263]
Rat glial cells	Glial cell cultures from rat basal forebrain	Semax	10 μM	-	BDNF mRNA ↑	-	-	-	[264]
Murine neuronal cells	Murine neuroblastoma Neuro2a cells	NDP-α-MSH	10–10,000 nM	-	BDNF mRNA ↑	-	-	-	[267]

↓: decrease; ↑: increase; ICV: intracerebroventricular; IP: intraperitoneal.

**Table 6:** A summary of the effects of melanocortin agonists on depression-like and anxious behavior.

Subjects	Experimental Model	Tests	Agonist	Dose	Site of Injection	Outcome	Agonist Effects Inhibitor	Dose	Site of Injection	Ref.
Healthy normal, male subjects	-	Tasks designed to assess emotionality or arousal	ACTH/MSH4-10	15 mg	IV	Anxiety ↓	-	-	-	[303]
Male SD rats	Social isolation induced anxiety- and depression-like behaviors	FST, EPM	-	-	-	Anxiety and depression-like behavior ↓	HS014 (solo effect)	1–10 nmol/rat	ICV	[312]
Male ICR mice, male Wistar rats, male SD rats	Conscious and freely moving mice and rats	EPM, light/dark exploration test, marble-burying behavior, learned helplessness test, FST	-	-	-	Anxiety and depression-like behavior ↓	MCL0129 (solo effect)	1–30 mg/kg	PO and SC	[313]
Male SD rats	Conscious and freely moving rats	Vogel test, EPM	-	-	-	Anxiety ↓	MCL0042 (solo effect)	1–10 mg/kg	SC	[314]
Male SD rats	Acute restraint and FST	EPM	-	-	-	Anxiety ↓	SHU 9119 (solo effect)	0.05, 0.5 nmol	ICV	[315]
Male SD rats	Conscious and freely moving rats and acute restraint stress	EPM	Cyclo (β-Ala-His-D-Phe-Arg-Trp-Glu)-NH2	0.1, 1 nmol	Medial amygdala	Anxiogenic-like effects of agonist and anxiolytic-like effect of antagonist	SHU 9119	0.5, 1.0 nmol	Medial amygdala	[316]
Male SD rats	Single prolonged stress	FST, EPM	-	-	-	Anxiety and depression-like behavior ↓	HS014 (solo effect)	3.5 ng, 100 μg	Intranasal	[317]
Male SD rats	Single prolonged stress	FST, EPM	-	-	-	Anxiety and depression-like behavior ↓	HS014 (solo effect)	3.5 ng, 100 μg	Intranasal	[318]
Male ICR mice, male SD rats	Stress-induced anxiogenic-like behavior	Vogel test, light/dark exploration test	α-MSH	3, 10 μg	ICV	Anxiogenic-like effects of agonists and anxiolytic-like effect of antagonist	MCL0020	0.01–0.1 nmol	ICV	[320]
			MT-II	0.1, 0.3, 1 μg						
Male SD rats	Ethanol-induced anxiolysis	EPM	α-MSH	0.5–5 μg/rat	ICV	Anxiogenic-like effects of agonist and anxiolytic-like effect of antagonist	HS014	1–10 nM/rat	ICV	[321]
							antiserum against α-MSH		ICV	
Male SD rats	Conscious and freely moving rats	Conflict test	ACTH1-24	0.1–10 μg	ICV	Anxiety ↑	-	-	-	[322]
			α-MSH	0.25–5 μg						
Female Wistar rats	Conscious and freely moving rats	EPM	α-MSH	100 ng	Medial preoptic area	Anxiety ↑	-	-	-	[323]
Male Wistar rats	IL-1β-induced anxiety-like behavior	EPM	α-MSH	0.2 μg	ICV	Anxiety ↓	HS014	2 μg	ICV	[325]
			γ-MSH	2 μg						
Male SD rats	Conscious and freely moving rats	FST	α-MSH	100–400 ng/rat	ICV	Antidepressant-like effect of antagonist and prodepressant effect of agonist	HS014	0.01–0.07 ng/rat	ICV	[326]
Male albino rats	Conscious and freely moving rats	FST	α-MSH	1 mg/kg	IP	Antidepressant-like effect	-	-	-	[328]
Male Wistar rats	Chronic immobilization stress-induced anxiety-like and depressive-like behaviour	FST, EPM	ACTH6-9-Pro-Gly-Pro	5, 50, 500 μg/kg	IP	Antidepressant and anxiolytic effects	-	-	-	[329]
Male SD rats	CUMS and LPS-induced depressive-like behaviour	Sucrose preference test	α-MSH	100 μg/kg	IP	Antidepressant effects	SHU 9119	4.3 μg/kg	IP	[330]
			ACTH4-10	58 μg/kg						
Male Wistar rats	Immobility-decreasing effect of imipramine and desipramine	FST	ACTH1-24	100 μg/day for 1, 3, 7, 14 days	SC	Prodepressant effect	-	-	-	[331]
Male Wistar rats	Immobility-decreasing effect of imipramine	FST	ACTH1-24	100 μg/day for 14 days	SC	Prodepressant effect	-	-	-	[332]
Male SD rats	Immobility-decreasing effect of imipramine	FST	ACTH1-24	100 μg/day for 14 days	IP	Prodepressant effect	-	-	-	[333]

↓: decrease; ↑: increase; SD: Sprague–Dawley rats; ICV: intracerebroventricular; IV: intravenous; SC: subcutaneous; IP: intraperitoneal; PO: peroral; FST: Porsolt’s forced swim test; EPM: elevated plus maze.

## Data Availability

Data are contained within the article.

## Abbreviations

ACTH: adrenocorticotropic hormone; AGRP: agouti-related protein; AMPAR: α-amino-3-hydroxy-5-methyl-4-isoxazolepropionic acid receptor; ASIP: agouti-signaling protein; AVP: arginine vasopressin; BDNF: brain-derived neurotrophic factor; CBR1: cannabinoid receptor type 1; COX-2: cyclooxygenase-2; CRH: corticotropin-releasing hormone; CUMS: chronic unpredictable mild stress; DEX: dexamethasone; ICV: intracerebroventricular; IL-6: interleukin-6; IL-1α: interleukin-1α; IL-1β: interleukin-1β; iNOS: inducible nitric oxide synthase; IP: intraperitoneal; IV: intravenous; GR: glucocorticoid receptor; HPAA: hypothalamic-pituitary-adrenal axis; LPS: lipopolysaccharide; MDD: major depressive disorder; α-MSH: α-melanocyte-stimulating hormone; MCRs: melanocortin receptors; MT-II: melanotan-II; NAc: nucleus accumbens; NGF: nerve growth factor; NMDAR: N-methyl-D-aspartate receptor; NSAIDs: non-steroidal anti-inflammatory drugs; NDP-α-MSH: [Nle4, D-Phe7]-α-melanocyte stimulating hormone; POMC: proopiomelanocortin; PVN: hypothalamic paraventricular nucleus; SC: subcutaneous; TNF-α: tumor necrosis factor-alpha; TRD: treatment-resistant depression; TrkB: tropomyosin receptor kinase B; VEGF: vascular endothelial growth factor; VTA: ventral tegmental area.

## References

[1] World Health Organization (2023). Depression. https://www.who.int/news-room/fact-sheets/detail/depression.

[2] Moitra M., Santomauro D., Collins P.Y., Vos T., Whiteford H., Saxena S., Ferrari A.J. (2022). The Global Gap in Treatment Coverage for Major Depressive Disorder in 84 Countries from 2000–2019: A Systematic Review and Bayesian Meta-Regression Analysis. PLoS Med..

[3] American Psychiatric Association (2013). Diagnostic and Statistical Manual of Mental Disorders.

[4] van Loo H.M., de Jonge P., Romeijn J.-W., Kessler R.C., Schoevers R.A. (2012). Data-Driven Subtypes of Major Depressive Disorder: A Systematic Review. BMC Med..

[5] Sullivan P.F., Neale M.C., Kendler K.S. (2000). Genetic Epidemiology of Major Depression: Review and Meta-Analysis. Am. J. Psychiatry.

[6] Kendler K.S., Gatz M., Gardner C.O., Pedersen N.L. (2006). A Swedish National Twin Study of Lifetime Major Depression. Am. J. Psychiatry.

[7] Ripke S., Wray N.R., Lewis C.M., Hamilton S.P., Weissman M.M., Breen G., Byrne E.M., Blackwood D.H.R., Boomsma D.I., Cichon S. (2013). A Mega-Analysis of Genome-Wide Association Studies for Major Depressive Disorder. Mol. Psychiatry.

[8] Wray N.R., Ripke S., Mattheisen M., Trzaskowski M., Byrne E.M., Abdellaoui A., Adams M.J., Agerbo E., Air T.M., Andlauer T.M.F. (2018). Genome-Wide Association Analyses Identify 44 Risk Variants and Refine the Genetic Architecture of Major Depression. Nat. Genet..

[9] Howard D.M., Adams M.J., Clarke T.-K., Hafferty J.D., Gibson J., Shirali M., Coleman J.R.I., Hagenaars S.P., Ward J., Wigmore E.M. (2019). Genome-Wide Meta-Analysis of Depression Identifies 102 Independent Variants and Highlights the Importance of the Prefrontal Brain Regions. Nat. Neurosci..

[10] Flint J. (2023). The Genetic Basis of Major Depressive Disorder. Mol. Psychiatry.

[11] Dall’Aglio L., Lewis C.M., Pain O. (2021). Delineating the Genetic Component of Gene Expression in Major Depression. Biol. Psychiatry.

[12] Fabbri C., Pain O., Hagenaars S.P., Lewis C.M., Serretti A. (2021). Transcriptome-Wide Association Study of Treatment-Resistant Depression and Depression Subtypes for Drug Repurposing. Neuropsychopharmacology.

[13] Li X., Su X., Liu J., Li H., Li M., Li W., Luo X.-J. (2021). Transcriptome-Wide Association Study Identifies New Susceptibility Genes and Pathways for Depression. Transl. Psychiatry.

[14] Mariani N., Cattane N., Pariante C., Cattaneo A. (2021). Gene Expression Studies in Depression Development and Treatment: An Overview of the Underlying Molecular Mechanisms and Biological Processes to Identify Biomarkers. Transl. Psychiatry.

[15] Morrison F.G., Miller M.W., Wolf E.J., Logue M.W., Maniates H., Kwasnik D., Cherry J.D., Svirsky S., Restaino A., Hildebrandt A. (2019). Reduced Interleukin 1A Gene Expression in the Dorsolateral Prefrontal Cortex of Individuals with PTSD and Depression. Neurosci. Lett..

[16] Leday G.G.R., Vértes P.E., Richardson S., Greene J.R., Regan T., Khan S., Henderson R., Freeman T.C., Pariante C.M., Harrison N.A. (2018). Replicable and Coupled Changes in Innate and Adaptive Immune Gene Expression in Two Case-Control Studies of Blood Microarrays in Major Depressive Disorder. Biol. Psychiatry.

[17] Cattaneo A., Ferrari C., Turner L., Mariani N., Enache D., Hastings C., Kose M., Lombardo G., McLaughlin A.P., Nettis M.A. (2020). Whole-Blood Expression of Inflammasome- and Glucocorticoid-Related MRNAs Correctly Separates Treatment-Resistant Depressed Patients from Drug-Free and Responsive Patients in the BIODEP Study. Transl. Psychiatry.

[18] Krishnan V., Nestler E.J. (2008). The Molecular Neurobiology of Depression. Nature.

[19] Li Z., Ruan M., Chen J., Fang Y. (2021). Major Depressive Disorder: Advances in Neuroscience Research and Translational Applications. Neurosci. Bull..

[20] Kamran M., Bibi F., ur Rehman A., Morris D.W. (2022). Major Depressive Disorder: Existing Hypotheses about Pathophysiological Mechanisms and New Genetic Findings. Genes.

[21] Lv S., Yao K., Zhang Y., Zhu S. (2023). NMDA Receptors as Therapeutic Targets for Depression Treatment: Evidence from Clinical to Basic Research. Neuropharmacology.

[22] Sheffler Z.M., Patel P., Abdijadid S. (2022). Antidepressants.

[23] Tian H., Hu Z., Xu J., Wang C. (2022). The Molecular Pathophysiology of Depression and the New Therapeutics. MedComm.

[24] Li K., Zhou G., Xiao Y., Gu J., Chen Q., Xie S., Wu J. (2022). Risk of Suicidal Behaviors and Antidepressant Exposure Among Children and Adolescents: A Meta-Analysis of Observational Studies. Front. Psychiatry.

[25] Fava M., Davidson K.G. (1996). Definition and epidemiology of treatment-resistant depression. Psychiatr. Clin. N. Am..

[26] Schroder H.S., Patterson E.H., Hirshbein L. (2022). Treatment-Resistant Depression Reconsidered. SSM–Ment. Health.

[27] Kirsch I., Deacon B.J., Huedo-Medina T.B., Scoboria A., Moore T.J., Johnson B.T. (2008). Initial Severity and Antidepressant Benefits: A Meta-Analysis of Data Submitted to the Food and Drug Administration. PLoS Med..

[28] Hengartner M.P., Jakobsen J.C., Sørensen A., Plöderl M. (2020). Efficacy of New-Generation Antidepressants Assessed with the Montgomery-Asberg Depression Rating Scale, the Gold Standard Clinician Rating Scale: A Meta-Analysis of Randomised Placebo-Controlled Trials. PLoS ONE.

[29] Moncrieff J., Cooper R.E., Stockmann T., Amendola S., Hengartner M.P., Horowitz M.A. (2022). The Serotonin Theory of Depression: A Systematic Umbrella Review of the Evidence. Mol. Psychiatry.

[30] Machado-Vieira R., Baumann J., Wheeler-Castillo C., Latov D., Henter I., Salvadore G., Zarate C. (2010). The Timing of Antidepressant Effects: A Comparison of Diverse Pharmacological and Somatic Treatments. Pharmaceuticals.

[31] Berman R.M., Cappiello A., Anand A., Oren D.A., Heninger G.R., Charney D.S., Krystal J.H. (2000). Antidepressant Effects of Ketamine in Depressed Patients. Biol. Psychiatry.

[32] aan het Rot M., Zarate C.A., Charney D.S., Mathew S.J. (2012). Ketamine for Depression: Where Do We Go from Here?. Biol. Psychiatry.

[33] Yavi M., Lee H., Henter I.D., Park L.T., Zarate C.A. (2022). Ketamine Treatment for Depression: A Review. Discov. Ment. Health.

[34] Williams N.R., Heifets B.D., Blasey C., Sudheimer K., Pannu J., Pankow H., Hawkins J., Birnbaum J., Lyons D.M., Rodriguez C.I. (2018). Attenuation of Antidepressant Effects of Ketamine by Opioid Receptor Antagonism. Am. J. Psychiatry.

[35] Zhang F., Hillhouse T.M., Anderson P.M., Koppenhaver P.O., Kegen T.N., Manicka S.G., Lane J.T., Pottanat E., Van Fossen M., Rice R. (2021). Opioid Receptor System Contributes to the Acute and Sustained Antidepressant-like Effects, but Not the Hyperactivity Motor Effects of Ketamine in Mice. Pharmacol. Biochem. Behav..

[36] Alldredge B. (2010). Pathogenic Involvement of Neuropeptides in Anxiety and Depression. Neuropeptides.

[37] Holmes A., Heilig M., Rupniak N.M.J., Steckler T., Griebel G. (2003). Neuropeptide Systems as Novel Therapeutic Targets for Depression and Anxiety Disorders. Trends Pharmacol. Sci..

[38] Kormos V., Gaszner B. (2013). Role of Neuropeptides in Anxiety, Stress, and Depression: From Animals to Humans. Neuropeptides.

[39] Kupcova I., Danisovic L., Grgac I., Harsanyi S. (2022). Anxiety and Depression: What Do We Know of Neuropeptides?. Behav. Sci..

[40] Lee M. (2007). The Central Melanocortin System and the Regulation of Energy Balance. Front. Biosci..

[41] Ulrich-Lai Y.M., Ryan K.K. (2014). Neuroendocrine Circuits Governing Energy Balance and Stress Regulation: Functional Overlap and Therapeutic Implications. Cell Metab..

[42] Micioni Di Bonaventura E., Botticelli L., Del Bello F., Giorgioni G., Piergentili A., Quaglia W., Romano A., Gaetani S., Micioni Di Bonaventura M.V., Cifani C. (2022). Investigating the Role of the Central Melanocortin System in Stress and Stress-Related Disorders. Pharmacol. Res..

[43] Laiho L., Murray J.F. (2022). The Multifaceted Melanocortin Receptors. Endocrinology.

[44] Harno E., Gali Ramamoorthy T., Coll A.P., White A. (2018). POMC: The Physiological Power of Hormone Processing. Physiol. Rev..

[45] Smith A.I., Funder J.W. (1988). Proopiomelanocortin Processing in the Pituitary, Central Nervous System, and Peripheral Tissues. Endocr. Rev..

[46] Jacobowitz D.M., O’Donohue T.L. (1978). Alpha-Melanocyte Stimulating Hormone: Immunohistochemical Identification and Mapping in Neurons of Rat Brain. Proc. Natl. Acad. Sci. USA.

[47] Wikberg J.E. (1999). Melanocortin Receptors: Perspectives for Novel Drugs. Eur. J. Pharmacol..

[48] Yang Y. (2011). Structure, Function and Regulation of the Melanocortin Receptors. Eur. J. Pharmacol..

[49] Getting S.J. (2006). Targeting Melanocortin Receptors as Potential Novel Therapeutics. Pharmacol. Ther..

[50] Shukla C., Koch L.G., Britton S.L., Cai M., Hruby V.J., Bednarek M., Novak C.M. (2015). Contribution of Regional Brain Melanocortin Receptor Subtypes to Elevated Activity Energy Expenditure in Lean, Active Rats. Neuroscience.

[51] Rodrigues A.R., Almeida H., Gouveia A.M. (2015). Intracellular Signaling Mechanisms of the Melanocortin Receptors: Current State of the Art. Cell Mol. Life Sci..

[52] Chan L.F., Webb T.R., Chung T.-T., Meimaridou E., Cooray S.N., Guasti L., Chapple J.P., Egertová M., Elphick M.R., Cheetham M.E. (2009). MRAP and MRAP2 Are Bidirectional Regulators of the Melanocortin Receptor Family. Proc. Natl. Acad. Sci. USA.

[53] Berruien N.N.A., Smith C.L. (2020). Emerging Roles of Melanocortin Receptor Accessory Proteins (MRAP and MRAP2) in Physiology and Pathophysiology. Gene.

[54] Novoselova T.V., Chan L.F., Clark A.J.L. (2018). Pathophysiology of Melanocortin Receptors and Their Accessory Proteins. Best Pract. Res. Clin. Endocrinol. Metab..

[55] Ericson M.D., Lensing C.J., Fleming K.A., Schlasner K.N., Doering S.R., Haskell-Luevano C. (2017). Bench-Top to Clinical Therapies: A Review of Melanocortin Ligands from 1954 to 2016. Biochim. Biophys. Acta–Mol. Basis Dis..

[56] Montero-Melendez T., Boesen T., Jonassen T.E.N. (2022). Translational Advances of Melanocortin Drugs: Integrating Biology, Chemistry and Genetics. Semin. Immunol..

[57] Ruhé H.G., Mason N.S., Schene A.H. (2007). Mood Is Indirectly Related to Serotonin, Norepinephrine and Dopamine Levels in Humans: A Meta-Analysis of Monoamine Depletion Studies. Mol. Psychiatry.

[58] Baumeister A.A., Hawkins M.F., Uzelac S.M. (2003). The Myth of Reserpine-Induced Depression: Role in the Historical Development of the Monoamine Hypothesis. J. Hist. Neurosci..

[59] Strawbridge R., Javed R.R., Cave J., Jauhar S., Young A.H. (2022). The Effects of Reserpine on Depression: A Systematic Review. J. Psychopharmacol..

[60] Haase J., Brown E. (2015). Integrating the Monoamine, Neurotrophin and Cytokine Hypotheses of Depression—A Central Role for the Serotonin Transporter?. Pharmacol. Ther..

[61] Sánchez M.S., Barontini M., Armando I., Celis M.E. (2001). Correlation of Increased Grooming Behavior and Motor Activity with Alterations in Nigrostriatal and Mesolimbic Catecholamines after Alpha-Melanotropin and Neuropeptide Glutamine-Isoleucine Injection in the Rat Ventral Tegmental Area. Cell Mol. Neurobiol..

[62] Davis J.F., Choi D.L., Shurdak J.D., Krause E.G., Fitzgerald M.F., Lipton J.W., Sakai R.R., Benoit S.C. (2011). Central Melanocortins Modulate Mesocorticolimbic Activity and Food Seeking Behavior in the Rat. Physiol. Behav..

[63] Roseberry A.G., Stuhrman K., Dunigan A.I. (2015). Regulation of the Mesocorticolimbic and Mesostriatal Dopamine Systems by α-Melanocyte Stimulating Hormone and Agouti-Related Protein. Neurosci. Biobehav. Rev..

[64] Lindblom J., Opmane B., Mutulis F., Mutule I., Petrovska R., Klusa V., Bergström L., Wikberg J.E.S. (2001). The MC4 Receptor Mediates α-MSH Induced Release of Nucleus Accumbens Dopamine. Neuroreport.

[65] Lindblom J., Kask A., Hägg E., Härmark L., Bergström L., Wikberg J. (2002). Chronic Infusion of a Melanocortin Receptor Agonist Modulates Dopamine Receptor Binding in the Rat Brain. Pharmacol. Res..

[66] Florijn W.J., De Boer T., Tonnaer J.A., Versteeg D.H. (1992). Characterization of the Inhibitory Effect of Adrenocorticotropin/Melanocyte-Stimulating Hormone-like Peptides on the Binding of Dopamine Receptor Ligands to the Dopamine D2 Receptor in Vitro. J. Pharmacol. Exp. Ther..

[67] Kawashima N., Chaki S., Okuyama S. (2003). Electrophysiological Effects of Melanocortin Receptor Ligands on Neuronal Activities of Monoaminergic Neurons in Rats. Neurosci. Lett..

[68] Markey K.A., Sze P.Y. (1984). Influence of ACTH on Tyrosine Hydroxylase Activity in the Locus Coeruleus of Mouse Brain. Neuroendocrinology.

[69] Versteeg D.H.G., Wurtman R.J. (1975). Effect of ACTH4–10 on the Rate of Synthesis of [3H]Catecholamines in the Brains of Intact, Hypophysectomized and Adrenalectomized Rats. Brain Res..

[70] Racca S., Spaccamiglio A., Esculapio P., Abbadessa G., Cangemi L., DiCarlo F., Portaleone P. (2005). Effects of Swim Stress and α-MSH Acute Pre-Treatment on Brain 5-HT Transporter and Corticosterone Receptor. Pharmacol. Biochem. Behav..

[71] Eremin K.O., Kudrin V.S., Saransaari P., Oja S.S., Grivennikov I.A., Myasoedov N.F., Rayevsky K.S. (2005). Semax, An ACTH(4-10) Analogue with Nootropic Properties, Activates Dopaminergic and Serotoninergic Brain Systems in Rodents. Neurochem. Res..

[72] Krishnadas R., Cavanagh J. (2012). Depression: An Inflammatory Illness? Figure 1. J. Neurol. Neurosurg. Psychiatry.

[73] Patel A. (2013). Review: The Role of Inflammation in Depression. Psychiatr. Danub..

[74] Brás J.P., Pinto S., Almeida M.I., Prata J., von Doellinger O., Coelho R., Barbosa M.A., Santos S.G. (2019). Peripheral Biomarkers of Inflammation in Depression: Evidence from Animal Models and Clinical Studies. Psychiatric Disorders: Methods and Protocols.

[75] Dowlati Y., Herrmann N., Swardfager W., Liu H., Sham L., Reim E.K., Lanctôt K.L. (2010). A Meta-Analysis of Cytokines in Major Depression. Biol. Psychiatry.

[76] Goldsmith D.R., Rapaport M.H., Miller B.J. (2016). A Meta-Analysis of Blood Cytokine Network Alterations in Psychiatric Patients: Comparisons between Schizophrenia, Bipolar Disorder and Depression. Mol. Psychiatry.

[77] Osimo E.F., Pillinger T., Rodriguez I.M., Khandaker G.M., Pariante C.M., Howes O.D. (2020). Inflammatory Markers in Depression: A Meta-Analysis of Mean Differences and Variability in 5,166 Patients and 5,083 Controls. Brain. Behav. Immun..

[78] Schaefer M., Engelbrechta M.A., Gut O., Fiebich B.L., Bauer J., Schmidt F., Grunze H., Lieb K. (2002). Interferon Alpha (IFNα) and Psychiatric Syndromes. Prog. Neuro-Psychopharmacol. Biol. Psychiatry.

[79] Schedlowski M., Engler H., Grigoleit J.-S. (2014). Endotoxin-Induced Experimental Systemic Inflammation in Humans: A Model to Disentangle Immune-to-Brain Communication. Brain. Behav. Immun..

[80] Reichenberg A., Yirmiya R., Schuld A., Kraus T., Haack M., Morag A., Pollmächer T. (2001). Cytokine-Associated Emotional and Cognitive Disturbances in Humans. Arch. Gen. Psychiatry.

[81] Eisenberger N.I., Inagaki T.K., Rameson L.T., Mashal N.M., Irwin M.R. (2009). An FMRI Study of Cytokine-Induced Depressed Mood and Social Pain: The Role of Sex Differences. Neuroimage.

[82] Grigoleit J.-S., Kullmann J.S., Wolf O.T., Hammes F., Wegner A., Jablonowski S., Engler H., Gizewski E., Oberbeck R., Schedlowski M. (2011). Dose-Dependent Effects of Endotoxin on Neurobehavioral Functions in Humans. PLoS ONE.

[83] Draper A., Koch R.M., van der Meer J.W., AJ Apps M., Pickkers P., Husain M., van der Schaaf M.E. (2018). Effort but Not Reward Sensitivity Is Altered by Acute Sickness Induced by Experimental Endotoxemia in Humans. Neuropsychopharmacology.

[84] Benson S., Rebernik L., Wegner A., Kleine-Borgmann J., Engler H., Schlamann M., Forsting M., Schedlowski M., Elsenbruch S. (2015). Neural Circuitry Mediating Inflammation-Induced Central Pain Amplification in Human Experimental Endotoxemia. Brain. Behav. Immun..

[85] Lasselin J., Elsenbruch S., Lekander M., Axelsson J., Karshikoff B., Grigoleit J.-S., Engler H., Schedlowski M., Benson S. (2016). Mood Disturbance during Experimental Endotoxemia: Predictors of State Anxiety as a Psychological Component of Sickness Behavior. Brain. Behav. Immun..

[86] Wegner A., Elsenbruch S., Maluck J., Grigoleit J.-S., Engler H., Jäger M., Spreitzer I., Schedlowski M., Benson S. (2014). Inflammation-Induced Hyperalgesia: Effects of Timing, Dosage, and Negative Affect on Somatic Pain Sensitivity in Human Experimental Endotoxemia. Brain. Behav. Immun..

[87] Grigoleit J.-S., Kullmann J.S., Oberbeck R., Schedlowski M., Engler H. (2013). Salivary α-Amylase Response to Endotoxin Administration in Humans. Psychoneuroendocrinology.

[88] Engler H., Benson S., Wegner A., Spreitzer I., Schedlowski M., Elsenbruch S. (2016). Men and Women Differ in Inflammatory and Neuroendocrine Responses to Endotoxin but Not in the Severity of Sickness Symptoms. Brain. Behav. Immun..

[89] Miller A.H., Maletic V., Raison C.L. (2009). Inflammation and Its Discontents: The Role of Cytokines in the Pathophysiology of Major Depression. Biol. Psychiatry.

[90] Berk M., Williams L.J., Jacka F.N., O’Neil A., Pasco J.A., Moylan S., Allen N.B., Stuart A.L., Hayley A.C., Byrne M.L. (2013). So Depression Is an Inflammatory Disease, but Where Does the Inflammation Come From?. BMC Med..

[91] Steptoe A., Kunz-Ebrecht S.R., Owen N. (2003). Lack of Association between Depressive Symptoms and Markers of Immune and Vascular Inflammation in Middle-Aged Men and Women. Psychol. Med..

[92] Himmerich H., Patsalos O., Lichtblau N., Ibrahim M.A.A., Dalton B. (2019). Cytokine Research in Depression: Principles, Challenges, and Open Questions. Front. Psychiatry.

[93] Szałach Ł.P., Lisowska K.A., Cubała W.J. (2019). The Influence of Antidepressants on the Immune System. Arch. Immunol. Ther. Exp..

[94] Kenis G., Maes M. (2002). Effects of Antidepressants on the Production of Cytokines. Int. J. Neuropsychopharmacol..

[95] Janssen D.G.A., Caniato R.N., Verster J.C., Baune B.T. (2010). A Psychoneuroimmunological Review on Cytokines Involved in Antidepressant Treatment Response. Hum. Psychopharmacol. Clin. Exp..

[96] Eyre H., Lavretsky H., Kartika J., Qassim A., Baune B. (2016). Modulatory Effects of Antidepressant Classes on the Innate and Adaptive Immune System in Depression. Pharmacopsychiatry.

[97] Dahl J., Ormstad H., Aass H.C.D., Malt U.F., Bendz L.T., Sandvik L., Brundin L., Andreassen O.A. (2014). The Plasma Levels of Various Cytokines Are Increased during Ongoing Depression and Are Reduced to Normal Levels after Recovery. Psychoneuroendocrinology.

[98] Strawbridge R., Arnone D., Danese A., Papadopoulos A., Herane Vives A., Cleare A.J. (2015). Inflammation and Clinical Response to Treatment in Depression: A Meta-Analysis. Eur. Neuropsychopharmacol..

[99] Köhler C.A., Freitas T.H., Stubbs B., Maes M., Solmi M., Veronese N., de Andrade N.Q., Morris G., Fernandes B.S., Brunoni A.R. (2017). Peripheral Alterations in Cytokine and Chemokine Levels After Antidepressant Drug Treatment for Major Depressive Disorder: Systematic Review and Meta-Analysis. Mol. Neurobiol..

[100] Więdłocha M., Marcinowicz P., Krupa R., Janoska-Jaździk M., Janus M., Dębowska W., Mosiołek A., Waszkiewicz N., Szulc A. (2018). Effect of Antidepressant Treatment on Peripheral Inflammation Markers–A Meta-Analysis. Prog. Neuro-Psychopharmacol. Biol. Psychiatry.

[101] Köhler O., Benros M.E., Nordentoft M., Farkouh M.E., Iyengar R.L., Mors O., Krogh J. (2014). Effect of Anti-Inflammatory Treatment on Depression, Depressive Symptoms, and Adverse Effects. JAMA Psychiatry.

[102] Eyre H.A., Air T., Proctor S., Rositano S., Baune B.T. (2015). A Critical Review of the Efficacy of Non-Steroidal Anti-Inflammatory Drugs in Depression. Prog. Neuro-Psychopharmacol. Biol. Psychiatry.

[103] Baune B.T. (2016). Are Non-Steroidal Anti-Inflammatory Drugs Clinically Suitable for the Treatment of Symptoms in Depression-Associated Inflammation?. Curr. Top. Behav. Neurosci..

[104] Warner-Schmidt J.L., Vanover K.E., Chen E.Y., Marshall J.J., Greengard P. (2011). Antidepressant Effects of Selective Serotonin Reuptake Inhibitors (SSRIs) Are Attenuated by Antiinflammatory Drugs in Mice and Humans. Proc. Natl. Acad. Sci. USA.

[105] Köhler-Forsberg O., Lydholm C.N., Hjorthøj C., Nordentoft M., Mors O., Benros M.E. (2019). Efficacy of Anti-inflammatory Treatment on Major Depressive Disorder or Depressive Symptoms: Meta-analysis of Clinical Trials. Acta Psychiatr. Scand..

[106] Zunszain P.A., Anacker C., Cattaneo A., Carvalho L.A., Pariante C.M. (2011). Glucocorticoids, Cytokines and Brain Abnormalities in Depression. Prog. Neuro-Psychopharmacol. Biol. Psychiatry.

[107] Beurel E., Toups M., Nemeroff C.B. (2020). The Bidirectional Relationship of Depression and Inflammation: Double Trouble. Neuron.

[108] Wang W., Guo D.-Y., Lin Y.-J., Tao Y.-X. (2019). Melanocortin Regulation of Inflammation. Front. Endocrinol..

[109] Dinparastisaleh R., Mirsaeidi M. (2021). Antifibrotic and Anti-Inflammatory Actions of α-Melanocytic Hormone: New Roles for an Old Player. Pharmaceuticals.

[110] Luger T.A., Scholzen T.E., Brzoska T., Böhm M. (2003). New Insights into the Functions of α-MSH and Related Peptides in the Immune System. Ann. N. Y. Acad. Sci..

[111] Maaser C., Kannengiesser K., Kucharzik T. (2006). Role of the Melanocortin System in Inflammation. Ann. N. Y. Acad. Sci..

[112] Brzoska T., Luger T.A., Maaser C., Abels C., Böhm M. (2008). α-Melanocyte-Stimulating Hormone and Related Tripeptides: Biochemistry, Antiinflammatory and Protective Effects in Vitro and in Vivo, and Future Perspectives for the Treatment of Immune-Mediated Inflammatory Diseases. Endocr. Rev..

[113] Martin L.W., Lipton J.M. (1990). Acute Phase Response to Endotoxin: Rise in Plasma Alpha-MSH and Effects of Alpha-MSH Injection. Am. J. Physiol. Integr. Comp. Physiol..

[114] Catania A., Suffredini A.F., Lipton J.M. (1995). Endotoxin Causes Release of α-Melanocyte-Stimulating Hormone in Normal Human Subjects. Neuroimmunomodulation.

[115] Sergeyev V., Broberger C., Hökfelt T. (2001). Effect of LPS Administration on the Expression of POMC, NPY, Galanin, CART and MCH MRNAs in the Rat Hypothalamus. Mol. Brain Res..

[116] Goelst K., Mitchell D., Laburn H. (1991). Effects of Alpha-Melanocyte Stimulating Hormone on Fever Caused by Endotoxin in Rabbits. J. Physiol..

[117] Martin L.W., Catania A., Hiltz M.E., Lipton J.M. (1991). Neuropeptide α-MSH Antagonizes IL-6- and TNF-Induced Fever. Peptides.

[118] Holdeman M., Lipton J.M. (1985). Antipyretic Activity of a Potent α-MSH Analog. Peptides.

[119] Huang Q.-H., Entwistle M.L., Alvaro J.D., Duman R.S., Hruby V.J., Tatro J.B. (1997). Antipyretic Role of Endogenous Melanocortins Mediated by Central Melanocortin Receptors during Endotoxin-Induced Fever. J. Neurosci..

[120] Huang Q.-H., Hruby V.J., Tatro J.B. (1998). Systemic α-MSH Suppresses LPS Fever via Central Melanocortin Receptors Independently of Its Suppression of Corticosterone and IL-6 Release. Am. J. Physiol. Integr. Comp. Physiol..

[121] Sinha P.S., Schiöth H.B., Tatro J.B. (2004). Roles of the Melanocortin-4 Receptor in Antipyretic and Hyperthermic Actions of Centrally Administered α-MSH. Brain Res..

[122] Lipton J.M., Catania A., Delgado R. (1998). Peptide Modulation of Inflammatory Processes within the Brain. Neuroimmunomodulation.

[123] Caruso C., Mohn C., Karara A.L., Rettori V., Watanobe H., Schiöth H.B., Seilicovich A., Lasaga M. (2004). Alpha-Melanocyte-Stimulating Hormone through Melanocortin-4 Receptor Inhibits Nitric Oxide Synthase and Cyclooxygenase Expression in the Hypothalamus of Male Rats. Neuroendocrinology.

[124] Rajora N., Boccoli G., Burns D., Sharma S., Catania A.P., Lipton J.M. (1997). α-MSH Modulates Local and Circulating Tumor Necrosis Factor-α in Experimental Brain Inflammation. J. Neurosci..

[125] Filippenkov I.B., Stavchansky V.V., Denisova A.E., Yuzhakov V.V., Sevan’kaeva L.E., Sudarkina O.Y., Dmitrieva V.G., Gubsky L.V., Myasoedov N.F., Limborska S.A. (2020). Novel Insights into the Protective Properties of ACTH(4-7)PGP (Semax) Peptide at the Transcriptome Level Following Cerebral Ischaemia–Reperfusion in Rats. Genes.

[126] Dergunova L.V., Dmitrieva V.G., Filippenkov I.B., Stavchansky V.V., Denisova A.E., Yuzhakov V.V., Sevan’kaeva L.E., Valieva L.V., Sudarkina O.Y., Gubsky L.V. (2021). The Peptide Drug ACTH(4-7)PGP (Semax) Suppresses mRNA Transcripts Encoding Proinflammatory Mediators Induced by Reversible Ischemia of the Rat Brain. Mol. Biol..

[127] Yu S., Doycheva D.M., Gamdzyk M., Yang Y., Lenahan C., Li G., Li D., Lian L., Tang J., Lu J. (2021). Activation of MC1R with BMS-470539 Attenuates Neuroinflammation via CAMP/PKA/Nurr1 Pathway after Neonatal Hypoxic-Ischemic Brain Injury in Rats. J. Neuroinflamm..

[128] Delgado R., Carlin A., Airaghi L., Demitri M.T., Meda L., Galimberti D., Baron P., Lipton J.M., Catania A. (1998). Melanocortin Peptides Inhibit Production of Proinflammatory Cytokines and Nitric Oxide by Activated Microglia. J. Leukoc. Biol..

[129] Yue Wong K., Rajora N., Boccoli G., Catania A., Lipton J.M. (1997). A Potential Mechanism of Local Anti-Inflammatory Action of Alpha-Melanocyte-Stimulating Hormone within the Brain: Modulation of Tumor Necrosis Factor-Alpha Production by Human Astrocytic Cells. Neuroimmunomodulation.

[130] Kamermans A., Verhoeven T., van het Hof B., Koning J.J., Borghuis L., Witte M., van Horssen J., de Vries H.E., Rijnsburger M. (2019). Setmelanotide, a Novel, Selective Melanocortin Receptor-4 Agonist Exerts Anti-Inflammatory Actions in Astrocytes and Promotes an Anti-Inflammatory Macrophage Phenotype. Front. Immunol..

[131] Lipton J.M., Catania A. (1998). Mechanisms of Antiinflammatory Action of the Neuroimmunomodulatory Peptide α-MSH. Ann. N. Y. Acad. Sci..

[132] Star R.A., Rajora N., Huang J., Stock R.C., Catania A., Lipton J.M. (1995). Evidence of Autocrine Modulation of Macrophage Nitric Oxide Synthase by Alpha-Melanocyte-Stimulating Hormone. Proc. Natl. Acad. Sci. USA.

[133] Neumann Andersen G., Nagaeva O., Mandrika I., Petrovska R., Muceniece R., Mincheva-Nilsson L., Wikberg J.E.S. (2002). MC1 Receptors Are Constitutively Expressed on Leucocyte Subpopulations with Antigen Presenting and Cytotoxic Functions. Clin. Exp. Immunol..

[134] Catania A., Rajora N., Capsoni F., Minonzio F., Star R.A., Lipton J.M. (1996). The Neuropeptide α-MSH Has Specific Receptors on Neutrophils and Reduces Chemotaxis in Vitro. Peptides.

[135] Becher E., Mahnke K., Brzoska T., Kalden D.-H., Grabbe S., Luger T.A. (2006). Human Peripheral Blood-Derived Dendritic Cells Express Functional Melanocortin Receptor MC-1R. Ann. N. Y. Acad. Sci..

[136] Johnson E.W., Hughes T.K., Smith E.M. (2001). ACTH Receptor Distribution and Modulation among Murine Mononuclear Leukocyte Populations. J. Biol. Regul. Homeost. Agents.

[137] Getting S.J., Gibbs L., Clark A.J., Flower R.J., Perretti M. (1999). POMC Gene-Derived Peptides Activate Melanocortin Type 3 Receptor on Murine Macrophages, Suppress Cytokine Release, and Inhibit Neutrophil Migration in Acute Experimental Inflammation. J. Immunol..

[138] BUGGY J.J. (1998). Binding of α-Melanocyte-Stimulating Hormone to Its G-Protein-Coupled Receptor on B-Lymphocytes Activates the Jak/STAT Pathway. Biochem. J..

[139] Andersen M., Nagaev I., Meyer M.K., Nagaeva O., Wikberg J., Mincheva-Nilsson L., Andersen G.N. (2017). Melanocortin 2, 3 and 4 Receptor Gene Expressions Are Downregulated in CD8^+^ T Cytotoxic Lymphocytes and CD19 ^+^ B Lymphocytes in Rheumatoid Arthritis Responding to TNF- *α* Inhibition. Scand. J. Immunol..

[140] Muceniece R., Dambrova M. (2010). Melanocortins in Brain Inflammation: The Role of Melanocortin Receptor Subtypes. Melanocortins: Multiple Actions and Therapeutic Potential.

[141] Kishi T., Aschkenasi C.J., Lee C.E., Mountjoy K.G., Saper C.B., Elmquist J.K. (2003). Expression of Melanocortin 4 Receptor MRNA in the Central Nervous System of the Rat. J. Comp. Neurol..

[142] Roselli-Rehfuss L., Mountjoy K.G., Robbins L.S., Mortrud M.T., Low M.J., Tatro J.B., Entwistle M.L., Simerly R.B., Cone R.D. (1993). Identification of a Receptor for Gamma Melanotropin and Other Proopiomelanocortin Peptides in the Hypothalamus and Limbic System. Proc. Natl. Acad. Sci. USA.

[143] Lam C., Getting S. (2004). Melanocortin Receptor Type 3 as a Potential Target for Anti-Inflammatory Therapy. Curr. Drug Target -Inflamm. Allergy.

[144] Lasaga M., Debeljuk L., Durand D., Scimonelli T.N., Caruso C. (2008). Role of α-Melanocyte Stimulating Hormone and Melanocortin 4 Receptor in Brain Inflammation. Peptides.

[145] Caruso C., Durand D., Schiöth H.B., Rey R., Seilicovich A., Lasaga M. (2007). Activation of Melanocortin 4 Receptors Reduces the Inflammatory Response and Prevents Apoptosis Induced by Lipopolysaccharide and Interferon-γ in Astrocytes. Endocrinology.

[146] Whitaker K.W., Reyes T.M. (2008). Central Blockade of Melanocortin Receptors Attenuates the Metabolic and Locomotor Responses to Peripheral Interleukin-1β Administration. Neuropharmacology.

[147] Cernackova A., Durackova Z., Trebaticka J., Mravec B. (2020). Neuroinflammation and Depressive Disorder: The Role of the Hypothalamus. J. Clin. Neurosci..

[148] Ceruso A., Martínez-Cengotitabengoa M., Peters-Corbett A., Diaz-Gutierrez M.J., Martínez-Cengotitabengoa M. (2020). Alterations of the HPA Axis Observed in Patients with Major Depressive Disorder and Their Relation to Early Life Stress: A Systematic Review. Neuropsychobiology.

[149] Mikulska J., Juszczyk G., Gawrońska-Grzywacz M., Herbet M. (2021). HPA Axis in the Pathomechanism of Depression and Schizophrenia: New Therapeutic Strategies Based on Its Participation. Brain Sci..

[150] Papadimitriou A., Priftis K.N. (2009). Regulation of the Hypothalamic-Pituitary-Adrenal Axis. Neuroimmunomodulation.

[151] Herman J.P., Ostrander M.M., Mueller N.K., Figueiredo H. (2005). Limbic System Mechanisms of Stress Regulation: Hypothalamo-Pituitary-Adrenocortical Axis. Prog. Neuro-Psychopharmacol. Biol. Psychiatry.

[152] Sapolsky R.M., Krey L.C., McEwen B.S. (1984). Glucocorticoid-Sensitive Hippocampal Neurons Are Involved in Terminating the Adrenocortical Stress Response. Proc. Natl. Acad. Sci. USA.

[153] Jacobson L., Sapolsky R. (1991). The Role of the Hippocampus in Feedback Regulation of the Hypothalamic-Pituitary-Adrenocortical Axis. Endocr. Rev..

[154] Cole A.B., Montgomery K., Bale T.L., Thompson S.M. (2022). What the Hippocampus Tells the HPA Axis: Hippocampal Output Attenuates Acute Stress Responses via Disynaptic Inhibition of CRF+ PVN Neurons. Neurobiol. Stress.

[155] Herman J., Schafer M., Young E., Thompson R., Douglass J., Akil H., Watson S. (1989). Evidence for Hippocampal Regulation of Neuroendocrine Neurons of the Hypothalamo-Pituitary-Adrenocortical Axis. J. Neurosci..

[156] Diorio D., Viau V., Meaney M. (1993). The Role of the Medial Prefrontal Cortex (Cingulate Gyrus) in the Regulation of Hypothalamic-Pituitary-Adrenal Responses to Stress. J. Neurosci..

[157] Feldman S., Conforti N., Itzik A., Weidenfeld J. (1994). Differential Effect of Amygdaloid Lesions on CRF-41, ACTH and Corticosterone Responses Following Neural Stimuli. Brain Res..

[158] Rubin R.T., Phillips J.J., McCracken J.T., Sadow T.F. (1996). Adrenal Gland Volume in Major Depression: Relationship to Basal and Stimulated Pituitary-Adrenal Cortical Axis Function. Biol. Psychiatry.

[159] Carroll B.J., Cassidy F., Naftolowitz D., Tatham N.E., Wilson W.H., Iranmanesh A., Liu P.Y., Veldhuis J.D. (2007). Pathophysiology of Hypercortisolism in Depression. Acta Psychiatr. Scand..

[160] Vreeburg S.A., Hoogendijk W.J.G., van Pelt J., DeRijk R.H., Verhagen J.C.M., van Dyck R., Smit J.H., Zitman F.G., Penninx B.W.J.H. (2009). Major Depressive Disorder and Hypothalamic-Pituitary-Adrenal Axis Activity. Arch. Gen. Psychiatry.

[161] Dienes K.A., Hazel N.A., Hammen C.L. (2013). Cortisol Secretion in Depressed, and at-Risk Adults. Psychoneuroendocrinology.

[162] Krishnan K.R.R., Doraiswamy P.M., Lurie S.N., Figiel G.S., Husain M.M., Boyko O.B., ELLINWOOD E.H., Nemeroff C.B. (1991). Pituitary Size in Depression*. J. Clin. Endocrinol. Metab..

[163] Delvecchio G., Altamura A.C., Soares J.C., Brambilla P. (2017). Pituitary Gland in Bipolar Disorder and Major Depression: Evidence from Structural MRI Studies. J. Affect. Disord..

[164] Wu T.-C., Chen H.-T., Chang H.-Y., Yang C.-Y., Hsiao M.-C., Cheng M.-L., Chen J.-C. (2013). Mineralocorticoid Receptor Antagonist Spironolactone Prevents Chronic Corticosterone Induced Depression-like Behavior. Psychoneuroendocrinology.

[165] Kvarta M.D., Bradbrook K.E., Dantrassy H.M., Bailey A.M., Thompson S.M. (2015). Corticosterone Mediates the Synaptic and Behavioral Effects of Chronic Stress at Rat Hippocampal Temporoammonic Synapses. J. Neurophysiol..

[166] Sterner E.Y., Kalynchuk L.E. (2010). Behavioral and Neurobiological Consequences of Prolonged Glucocorticoid Exposure in Rats: Relevance to Depression. Prog. Neuro-Psychopharmacol. Biol. Psychiatry.

[167] Ding H., Cui X.-Y., Cui S.-Y., Ye H., Hu X., Zhao H.-L., Liu Y.-T., Zhang Y.-H. (2018). Depression-like Behaviors Induced by Chronic Corticosterone Exposure via Drinking Water: Time-Course Analysis. Neurosci. Lett..

[168] Watson S., Gallagher P., Del-Estal D., Hearn A., Ferrier I.N., Young A.H. (2002). Hypothalamic–Pituitary–Adrenal Axis Function in Patients with Chronic Depression. Psychol. Med..

[169] Burke H.M., Davis M.C., Otte C., Mohr D.C. (2005). Depression and Cortisol Responses to Psychological Stress: A Meta-Analysis. Psychoneuroendocrinology.

[170] Knorr U., Vinberg M., Kessing L.V., Wetterslev J. (2010). Salivary Cortisol in Depressed Patients versus Control Persons: A Systematic Review and Meta-Analysis. Psychoneuroendocrinology.

[171] Carroll B.J., Iranmanesh A., Keenan D.M., Cassidy F., Wilson W.H., Veldhuis J.D. (2012). Pathophysiology of Hypercortisolism in Depression: Pituitary and Adrenal Responses to Low Glucocorticoid Feedback. Acta Psychiatr. Scand..

[172] Stokes P.E. (1984). Pretreatment DST and Hypothalamic-Pituitary-Adrenocortical Function in Depressed Patients and Comparison Groups. Arch. Gen. Psychiatry.

[173] Herbert J. (2013). Cortisol and Depression: Three Questions for Psychiatry. Psychol. Med..

[174] Carpenter W.T., Bunney W.E. (1971). Adrenal Cortical Activity in Depressive Illness. Am. J. Psychiatry.

[175] Nandam L.S., Brazel M., Zhou M., Jhaveri D.J. (2020). Cortisol and Major Depressive Disorder—Translating Findings from Humans to Animal Models and Back. Front. Psychiatry.

[176] Pariante C.M., Miller A.H. (2001). Glucocorticoid Receptors in Major Depression: Relevance to Pathophysiology and Treatment. Biol. Psychiatry.

[177] Pace T.W.W., Hu F., Miller A.H. (2007). Cytokine-Effects on Glucocorticoid Receptor Function: Relevance to Glucocorticoid Resistance and the Pathophysiology and Treatment of Major Depression. Brain. Behav. Immun..

[178] Ising M., Künzel H.E., Binder E.B., Nickel T., Modell S., Holsboer F. (2005). The Combined Dexamethasone/CRH Test as a Potential Surrogate Marker in Depression. Prog. Neuro-Psychopharmacol. Biol. Psychiatry.

[179] Watson S., Gallagher P., Smith M.S., Ferrier I.N., Young A.H. (2006). The Dex/CRH Test—Is It Better than the DST?. Psychoneuroendocrinology.

[180] Mokhtari M., Arfken C., Boutros N. (2013). The DEX/CRH Test for Major Depression: A Potentially Useful Diagnostic Test. Psychiatry Res..

[181] Holsboer F., Barden N. (1996). Antidepressants and Hypothalamic-Pituitary-Adrenocortical Regulation. Endocr. Rev..

[182] Mason B.L., Pariante C.M. (2006). The Effects of Antidepressants on the Hypothalamic-Pituitary-Adrenal Axis. Drug News Perspect..

[183] Horstmann S., Dose T., Lucae S., Kloiber S., Menke A., Hennings J., Spieler D., Uhr M., Holsboer F., Ising M. (2009). Suppressive Effect of Mirtazapine on the HPA System in Acutely Depressed Women Seems to Be Transient and Not Related to Antidepressant Action. Psychoneuroendocrinology.

[184] Menke A. (2019). Is the HPA Axis as Target for Depression Outdated, or Is There a New Hope?. Front. Psychiatry.

[185] Ding Y., Wei Z., Yan H., Guo W. (2021). Efficacy of Treatments Targeting Hypothalamic-Pituitary-Adrenal Systems for Major Depressive Disorder: A Meta-Analysis. Front. Pharmacol..

[186] Blasey C.M., DeBattista C., Roe R., Block T., Belanoff J.K. (2009). A Multisite Trial of Mifepristone for the Treatment of Psychotic Depression: A Site-by-Treatment Interaction. Contemp. Clin. Trials.

[187] Kling M.A., Coleman V.H., Schulkin J. (2009). Glucocorticoid Inhibition in the Treatment of Depression: Can We Think Outside the Endocrine Hypothalamus?. Depress. Anxiety.

[188] Lombardo G., Enache D., Gianotti L., Schatzberg A.F., Young A.H., Pariante C.M., Mondelli V. (2019). Baseline Cortisol and the Efficacy of Antiglucocorticoid Treatment in Mood Disorders: A Meta-Analysis. Psychoneuroendocrinology.

[189] Calogero A.E., Gallucci W.T., Gold P.W., Chrousos G.P. (1988). Multiple Feedback Regulatory Loops upon Rat Hypothalamic Corticotropin-Releasing Hormone Secretion. Potential Clinical Implications. J. Clin. Investig..

[190] Motta M., Mangili G., Martini L. (1965). A “Short” Feedback Loop in the Control of ACTH Secretion. Endocrinology.

[191] Sawchenko P.E., Arias C. (1995). Evidence for Short-Loop Feedback Effects of ACTH on CRF and Vasopressin Expression in Parvocellular Neurosecretory Neurons. J. Neuroendocrinol..

[192] Seiden G., Brodish A. (1971). Physiological Evidence for ‘Short-Loop’ Feedback Effects of ACTH on Hypothalamic CRF. Neuroendocrinology.

[193] Suda T., Yajima F., Tomori N., Sumitomo T., Nakagami Y., Ushiyama T., Demura H., Shizume K. (1986). Inhibitory effect of adrenocorticotropin on corticotropin-releasing factor release from rat hypothalamus in vitro. Endocrinology.

[194] Tozawa F., Suda T., Dobashi I., Ohmori N., Kasagi Y., Demura H. (1994). Central Administration of α-Melanocyte-Stimulating Hormone Inhibits Corticotropin-Releasing Factor Release in Adrenalectomized Rats. Neurosci. Lett..

[195] Schioth H., Muceniece R., Larsson M., Wikberg J. (1997). The Melanocortin 1, 3, 4 or 5 Receptors Do Not Have a Binding Epitope for ACTH beyond the Sequence of Alpha-MSH. J. Endocrinol..

[196] Lu X.-Y., Barsh G.S., Akil H., Watson S.J. (2003). Interaction between α-Melanocyte-Stimulating Hormone and Corticotropin-Releasing Hormone in the Regulation of Feeding and Hypothalamo-Pituitary-Adrenal Responses. J. Neurosci..

[197] Wiegant V.M., Jolles J., Colbern D.L., Zimmermann E., Hendrik Gispen W. (1979). Intracerebroventricular Acth Activates the Pituitary-Adrenal System:Dissociation from a Behavioral Response. Life Sci..

[198] Von Frijtag J.C., Croiset G., Hendrik Gispen W., Adan R.A.H., Wiegant V.M. (1998). The Role of Central Melanocortin Receptors in the Activation of the Hypothalamus-Pituitary-Adrenal-Axis and the Induction of Excessive Grooming. Br. J. Pharmacol..

[199] de Vries K. (2011). Brain Melanocortin Receptors Are Involved In CRH-Mediated HPA Axis Activity and Thermogenesis. Open Neuroendocrinol. J..

[200] Ryan K.K., Mul J.D., Clemmensen C., Egan A.E., Begg D.P., Halcomb K., Seeley R.J., Herman J.P., Ulrich-Lai Y.M. (2014). Loss of Melanocortin-4 Receptor Function Attenuates HPA Responses to Psychological Stress. Psychoneuroendocrinology.

[201] Serova L.I., Laukova M., Alaluf L.G., Sabban E.L. (2014). Blockage of Melanocortin-4 Receptors by Intranasal HS014 Attenuates Single Prolonged Stress-Triggered Changes in Several Brain Regions. J. Neurochem..

[202] Vecsernyés M., Biró É., Gardi J., Julesz J., Telegdy G. (2000). Involvement of Endogenous Corticotropin-Releasing Factor in Mediation of Neuroendocrine and Behavioral Effects to Alpha-Melanocyte-Stimulating Hormone. Endocr. Res..

[203] Weiss J.M., Sundar S.K., Cierpial M.A., Ritchie J.C. (1991). Effects of Interleukin-1 Infused into Brain Are Antagonized by α-MSH in a Dose-Dependent Manner. Eur. J. Pharmacol..

[204] Shalts E., Feng Y.J., Ferin M., Wardlaw S.L. (1992). Alpha-Melanocyte-Stimulating Hormone Antagonizes the Neuroendocrine Effects of Corticotropin-Releasing Factor and Interleukin-1 Alpha in the Primate. Endocrinology.

[205] Rivier C., Chizzonite R., Vale W. (1989). In the Mouse, the Activation of the Hypothalamic Pituitary-Adrenal Axis by a Lipopolysaccharide (Endotoxin) Is Mediated through Interleukin-1. Endocrinology.

[206] Daynes R.A., Robertson B.A., Cho B.H., Burnham D.K., Newton R. (1987). Alpha-Melanocyte-Stimulating Hormone Exhibits Target Cell Selectivity in Its Capacity to Affect Interleukin 1-Inducible Responses in Vivo and in Vitro. J. Immunol..

[207] Lyson K., McCann S.M. (1993). Alpha-Melanocyte-Stimulating Hormone Abolishes IL-1- and IL-6-Lnduced Corticotropin-Releasing Factor Release from the Hypothalamus in Vitro. Neuroendocrinology.

[208] Cragnolini A.B., Perelló M., Schiöth H.B., Scimonelli T.N. (2004). α-MSH and γ-MSH Inhibit IL-1β Induced Activation of the Hypothalamic–Pituitary–Adrenal Axis through Central Melanocortin Receptors. Regul. Pept..

[209] Vulliémoz N.R., Xiao E., Xia-Zhang L., Ferin M., Wardlaw S.L. (2006). Melanocortin Modulation of Inflammatory Cytokine and Neuroendocrine Responses to Endotoxin in the Monkey. Endocrinology.

[210] Li Y., Li F., Qin D., Chen H., Wang J., Wang J., Song S., Wang C., Wang Y., Liu S. (2022). The Role of Brain Derived Neurotrophic Factor in Central Nervous System. Front. Aging Neurosci..

[211] Wang C.S., Kavalali E.T., Monteggia L.M. (2022). BDNF Signaling in Context: From Synaptic Regulation to Psychiatric Disorders. Cell.

[212] Duman R.S., Monteggia L.M. (2006). A Neurotrophic Model for Stress-Related Mood Disorders. Biol. Psychiatry.

[213] Xue Y., Liang H., Yang R., Deng K., Tang M., Zhang M. (2021). The Role of Pro- and Mature Neurotrophins in the Depression. Behav. Brain Res..

[214] Castrén E., Monteggia L.M. (2021). Brain-Derived Neurotrophic Factor Signaling in Depression and Antidepressant Action. Biol. Psychiatry.

[215] Karege F., Perret G., Bondolfi G., Schwald M., Bertschy G., Aubry J.-M. (2002). Decreased Serum Brain-Derived Neurotrophic Factor Levels in Major Depressed Patients. Psychiatry Res..

[216] Molendijk M.L., Bus B.A.A., Spinhoven P., Penninx B.W.J.H., Kenis G., Prickaerts J., Voshaar R.O., Elzinga B.M. (2011). Serum Levels of Brain-Derived Neurotrophic Factor in Major Depressive Disorder: State–Trait Issues, Clinical Features and Pharmacological Treatment. Mol. Psychiatry.

[217] Shimizu E., Hashimoto K., Okamura N., Koike K., Komatsu N., Kumakiri C., Nakazato M., Watanabe H., Shinoda N., Okada S. (2003). Alterations of Serum Levels of Brain-Derived Neurotrophic Factor (BDNF) in Depressed Patients with or without Antidepressants. Biol. Psychiatry.

[218] Aydemir O., Deveci A., Taneli F. (2005). The Effect of Chronic Antidepressant Treatment on Serum Brain-Derived Neurotrophic Factor Levels in Depressed Patients: A Preliminary Study. Prog. Neuro-Psychopharmacol. Biol. Psychiatry.

[219] Gervasoni N., Aubry J.-M., Bondolfi G., Osiek C., Schwald M., Bertschy G., Karege F. (2005). Partial Normalization of Serum Brain-Derived Neurotrophic Factor in Remitted Patients after a Major Depressive Episode. Neuropsychobiology.

[220] Tadić A., Wagner S., Schlicht K.F., Peetz D., Borysenko L., Dreimüller N., Hiemke C., Lieb K. (2011). The Early Non-Increase of Serum BDNF Predicts Failure of Antidepressant Treatment in Patients with Major Depression: A Pilot Study. Prog. Neuro-Psychopharmacol. Biol. Psychiatry.

[221] Sen S., Duman R., Sanacora G. (2008). Serum Brain-Derived Neurotrophic Factor, Depression, and Antidepressant Medications: Meta-Analyses and Implications. Biol. Psychiatry.

[222] Kang H.-J., Kim J.-M., Lee J.-Y., Kim S.-Y., Bae K.-Y., Kim S.-W., Shin I.-S., Kim H.-R., Shin M.-G., Yoon J.-S. (2013). BDNF Promoter Methylation and Suicidal Behavior in Depressive Patients. J. Affect. Disord..

[223] Dunham J.S., Deakin J.F.W., Miyajima F., Payton A., Toro C.T. (2009). Expression of Hippocampal Brain-Derived Neurotrophic Factor and Its Receptors in Stanley Consortium Brains. J. Psychiatr. Res..

[224] Ray M.T., Shannon Weickert C., Webster M.J. (2014). Decreased BDNF and TrkB MRNA Expression in Multiple Cortical Areas of Patients with Schizophrenia and Mood Disorders. Transl. Psychiatry.

[225] Karege F., Vaudan G., Schwald M., Perroud N., La Harpe R. (2005). Neurotrophin Levels in Postmortem Brains of Suicide Victims and the Effects of Antemortem Diagnosis and Psychotropic Drugs. Mol. Brain Res..

[226] Chen B., Dowlatshahi D., MacQueen G.M., Wang J.-F., Young L.T. (2001). Increased Hippocampal Bdnf Immunoreactivity in Subjects Treated with Antidepressant Medication. Biol. Psychiatry.

[227] Hamani C., Machado D.C., Hipólide D.C., Dubiela F.P., Suchecki D., Macedo C.E., Tescarollo F., Martins U., Covolan L., Nobrega J.N. (2012). Deep Brain Stimulation Reverses Anhedonic-Like Behavior in a Chronic Model of Depression: Role of Serotonin and Brain Derived Neurotrophic Factor. Biol. Psychiatry.

[228] Jiang Y., Zhu J. (2015). Effects of Sleep Deprivation on Behaviors and Abnormal Hippocampal BDNF/MiR-10B Expression in Rats with Chronic Stress Depression. Int. J. Clin. Exp. Pathol..

[229] Taliaz D., Loya A., Gersner R., Haramati S., Chen A., Zangen A. (2011). Resilience to Chronic Stress Is Mediated by Hippocampal Brain-Derived Neurotrophic Factor. J. Neurosci..

[230] Toth E., Gersner R., Wilf-Yarkoni A., Raizel H., Dar D.E., Richter-Levin G., Levit O., Zangen A. (2008). Age-Dependent Effects of Chronic Stress on Brain Plasticity and Depressive Behavior. J. Neurochem..

[231] Schnydrig S., Korner L., Landweer S., Ernst B., Walker G., Otten U., Kunz D. (2007). Peripheral Lipopolysaccharide Administration Transiently Affects Expression of Brain-Derived Neurotrophic Factor, Corticotropin and Proopiomelanocortin in Mouse Brain. Neurosci. Lett..

[232] Lapchak P.A., Araujo D.M., Hefti F. (1993). Systemic Interleukin-1β Decreases Brain-Derived Neurotrophic Factor Messenger RNA Expression in the Rat Hippocampal Formation. Neuroscience.

[233] Guan Z., Fang J. (2006). Peripheral Immune Activation by Lipopolysaccharide Decreases Neurotrophins in the Cortex and Hippocampus in Rats. Brain. Behav. Immun..

[234] Taliaz D., Stall N., Dar D.E., Zangen A. (2010). Knockdown of Brain-Derived Neurotrophic Factor in Specific Brain Sites Precipitates Behaviors Associated with Depression and Reduces Neurogenesis. Mol. Psychiatry.

[235] Hoshaw B.A., Malberg J.E., Lucki I. (2005). Central Administration of IGF-I and BDNF Leads to Long-Lasting Antidepressant-like Effects. Brain Res..

[236] Siuciak J.A., Lewis D.R., Wiegand S.J., Lindsay R.M. (1997). Antidepressant-Like Effect of Brain-Derived Neurotrophic Factor (BDNF). Pharmacol. Biochem. Behav..

[237] Shirayama Y., Chen A.C.-H., Nakagawa S., Russell D.S., Duman R.S. (2002). Brain-Derived Neurotrophic Factor Produces Antidepressant Effects in Behavioral Models of Depression. J. Neurosci..

[238] Nibuya M., Morinobu S., Duman R. (1995). Regulation of BDNF and TrkB MRNA in Rat Brain by Chronic Electroconvulsive Seizure and Antidepressant Drug Treatments. J. Neurosci..

[239] Coppell A., Pei Q., Zetterström T.S. (2003). Bi-Phasic Change in BDNF Gene Expression Following Antidepressant Drug Treatment. Neuropharmacology.

[240] Jacobsen J.P.R., Mørk A. (2004). The Effect of Escitalopram, Desipramine, Electroconvulsive Seizures and Lithium on Brain-Derived Neurotrophic Factor MRNA and Protein Expression in the Rat Brain and the Correlation to 5-HT and 5-HIAA Levels. Brain Res..

[241] Calabrese F., Molteni R., Maj P.F., Cattaneo A., Gennarelli M., Racagni G., Riva M.A. (2007). Chronic Duloxetine Treatment Induces Specific Changes in the Expression of BDNF Transcripts and in the Subcellular Localization of the Neurotrophin Protein. Neuropsychopharmacology.

[242] Casarotto P.C., Girych M., Fred S.M., Kovaleva V., Moliner R., Enkavi G., Biojone C., Cannarozzo C., Sahu M.P., Kaurinkoski K. (2021). Antidepressant Drugs Act by Directly Binding to TRKB Neurotrophin Receptors. Cell.

[243] Casarotto P., Umemori J., Castrén E. (2022). BDNF Receptor TrkB as the Mediator of the Antidepressant Drug Action. Front. Mol. Neurosci..

[244] Alboni S., Tascedda F., Corsini D., Benatti C., Caggia F., Capone G., Barden N., Blom J.M.C., Brunello N. (2011). Stress Induces Altered CRE/CREB Pathway Activity and BDNF Expression in the Hippocampus of Glucocorticoid Receptor-Impaired Mice. Neuropharmacology.

[245] Ridder S., Chourbaji S., Hellweg R., Urani A., Zacher C., Schmid W., Zink M., Hörtnagl H., Flor H., Henn F.A. (2005). Mice with Genetically Altered Glucocorticoid Receptor Expression Show Altered Sensitivity for Stress-Induced Depressive Reactions. J. Neurosci..

[246] Numakawa T., Adachi N., Richards M., Chiba S., Kunugi H. (2013). Brain-Derived Neurotrophic Factor and Glucocorticoids: Reciprocal Influence on the Central Nervous System. Neuroscience.

[247] Schaaf M.J., Hoetelmans R.W., de Kloet E.R., Vreugdenhil E. (1997). Corticosterone Regulates Expression of BDNF and TrkB but Not NT-3 and TrkC MRNA in the Rat Hippocampus. J. Neurosci. Res..

[248] Chao H.M., Sakai R.R., Ma L.Y., McEwen B.S. (1998). Adrenal Steroid Regulation of Neurotrophic Factor Expression in the Rat Hippocampus. Endocrinology.

[249] Hansson A.C., Cintra A., Belluardo N., Sommer W., Bhatnagar M., Bader M., Ganten D., Fuxe K. (2000). Gluco- and Mineralocorticoid Receptor-Mediated Regulation of Neurotrophic Factor Gene Expression in the Dorsal Hippocampus and the Neocortex of the Rat. Eur. J. Neurosci..

[250] Lambert W.M., Xu C.-F., Neubert T.A., Chao M.V., Garabedian M.J., Jeanneteau F.D. (2013). Brain-Derived Neurotrophic Factor Signaling Rewrites the Glucocorticoid Transcriptome via Glucocorticoid Receptor Phosphorylation. Mol. Cell Biol..

[251] Jeanneteau F., Garabedian M.J., Chao M.V. (2008). Activation of Trk Neurotrophin Receptors by Glucocorticoids Provides a Neuroprotective Effect. Proc. Natl. Acad. Sci. USA.

[252] Dolotov O.V., Karpenko E.A., Inozemtseva L.S., Seredenina T.S., Levitskaya N.G., Rozyczka J., Dubynina E.V., Novosadova E.V., Andreeva L.A., Alfeeva L.Y. (2006). Semax, an Analog of ACTH(4–10) with Cognitive Effects, Regulates BDNF and TrkB Expression in the Rat Hippocampus. Brain Res..

[253] Shadrina M., Kolomin T., Agapova T., Agniullin Y., Shram S., Slominsky P., Lymborska S., Myasoedov N. (2010). Comparison of the Temporary Dynamics of NGF and BDNF Gene Expression in Rat Hippocampus, Frontal Cortex, and Retina Under Semax Action. J. Mol. Neurosci..

[254] Nicholson J.R., Peter J.-C., Lecourt A.-C., Barde Y.-A., Hofbauer K.G. (2007). Melanocortin-4 Receptor Activation Stimulates Hypothalamic Brain-Derived Neurotrophic Factor Release to Regulate Food Intake, Body Temperature and Cardiovascular Function. J. Neuroendocrinol..

[255] Xu B., Goulding E.H., Zang K., Cepoi D., Cone R.D., Jones K.R., Tecott L.H., Reichardt L.F. (2003). Brain-Derived Neurotrophic Factor Regulates Energy Balance Downstream of Melanocortin-4 Receptor. Nat. Neurosci..

[256] Hohenadel M.G., Thearle M.S., Grice B.A., Huang H., Dai M.-H., Tao Y.-X., Hunter L.A., Palaguachi G.I., Mou Z., Kim R.C. (2014). Brain-Derived Neurotrophic Factor in Human Subjects with Function-Altering Melanocortin-4 Receptor Variants. Int. J. Obes..

[257] Bariohay B., Roux J., Tardivel C., Trouslard J., Jean A., Lebrun B. (2009). Brain-Derived Neurotrophic Factor/Tropomyosin-Related Kinase Receptor Type B Signaling Is a Downstream Effector of the Brainstem Melanocortin System in Food Intake Control. Endocrinology.

[258] Saba J., Carniglia L., Ramírez D., Turati J., Imsen M., Durand D., Lasaga M., Caruso C. (2019). Melanocortin 4 Receptor Activation Protects Striatal Neurons and Glial Cells from 3-Nitropropionic Acid Toxicity. Mol. Cell Neurosci..

[259] Dolotov O.V., Karpenko E.A., Seredenina T.S., Inozemtseva L.S., Levitskaya N.G., Zolotarev Y.A., Kamensky A.A., Grivennikov I.A., Engele J., Myasoedov N.F. (2006). Semax, an Analogue of Adrenocorticotropin (4–10), Binds Specifically and Increases Levels of Brain-Derived Neurotrophic Factor Protein in Rat Basal Forebrain. J. Neurochem..

[260] Liao Y., Xing Q., Li Q., Zhang J., Pan R., Yuan Z. (2021). Astrocytes in Depression and Alzheimer’s Disease. Front. Med..

[261] Dolotov O.V., Inozemtseva L.S., Myasoedov N.F., Grivennikov I.A. (2022). Stress-Induced Depression and Alzheimer’s Disease: Focus on Astrocytes. Int. J. Mol. Sci..

[262] Caruso C., Carniglia L., Durand D., Gonzalez P.V., Scimonelli T.N., Lasaga M. (2012). Melanocortin 4 Receptor Activation Induces Brain-Derived Neurotrophic Factor Expression in Rat Astrocytes through Cyclic AMP–Protein Kinase A Pathway. Mol. Cell Endocrinol..

[263] Ramírez D., Saba J., Carniglia L., Durand D., Lasaga M., Caruso C. (2015). Melanocortin 4 Receptor Activates ERK-CFos Pathway to Increase Brain-Derived Neurotrophic Factor Expression in Rat Astrocytes and Hypothalamus. Mol. Cell Endocrinol..

[264] Shadrina M.I., Dolotov O.V., Grivennikov I.A., Slominsky P.A., Andreeva L.A., Inozemtseva L.S., Limborska S.A., Myasoedov N.F. (2001). Rapid Induction of Neurotrophin MRNAs in Rat Glial Cell Cultures by Semax, an Adrenocorticotropic Hormone Analog. Neurosci. Lett..

[265] Dubynina E.V., Inozemtseva L.S., Markov D.D., Yatsenko K.A., Dolotov O.V., Grivennikov I.A. (2009). Alpha-Melanocyte-Stimulating Hormone Increases the Expression of Vascular Endothelial Growth Factor in Rat Hippocampal Astrocytes in Vitro. Neurochem. J..

[266] Deyama S., Bang E., Kato T., Li X.-Y., Duman R.S. (2019). Neurotrophic and Antidepressant Actions of Brain-Derived Neurotrophic Factor Require Vascular Endothelial Growth Factor. Biol. Psychiatry.

[267] Zhang W., Wu Y. (2020). Mitogen- and Stress-Activated Protein Kinase-1 Activation Is Involved in Melanocortin-Induced BDNF Expression in Neuro2a Neuronal Cells. Neuroreport.

[268] Samuels B.A., Hen R. (2011). Neurogenesis and Affective Disorders. Eur. J. Neurosci..

[269] Campbell S., Marriott M., Nahmias C., MacQueen G.M. (2004). Lower Hippocampal Volume in Patients Suffering from Depression: A Meta-Analysis. Am. J. Psychiatry.

[270] Videbech P. (2004). Hippocampal Volume and Depression: A Meta-Analysis of MRI Studies. Am. J. Psychiatry.

[271] McKinnon M.C., Yucel K., Nazarov A., MacQueen G.M. (2009). A Meta-Analysis Examining Clinical Predictors of Hippocampal Volume in Patients with Major Depressive Disorder. J. Psychiatry Neurosci..

[272] Cole J., Costafreda S.G., McGuffin P., Fu C.H.Y. (2011). Hippocampal Atrophy in First Episode Depression: A Meta-Analysis of Magnetic Resonance Imaging Studies. J. Affect. Disord..

[273] Nolan M., Roman E., Nasa A., Levins K.J., O’Hanlon E., O’Keane V., Willian Roddy D. (2020). Hippocampal and Amygdalar Volume Changes in Major Depressive Disorder: A Targeted Review and Focus on Stress. Chronic Stress.

[274] Koolschijn P.C.M.P., van Haren N.E.M., Lensvelt-Mulders G.J.L.M., Hulshoff Pol H.E., Kahn R.S. (2009). Brain Volume Abnormalities in Major Depressive Disorder: A Meta-Analysis of Magnetic Resonance Imaging Studies. Hum. Brain Mapp..

[275] Surget A., Saxe M., Leman S., Ibarguen-Vargas Y., Chalon S., Griebel G., Hen R., Belzung C. (2008). Drug-Dependent Requirement of Hippocampal Neurogenesis in a Model of Depression and of Antidepressant Reversal. Biol. Psychiatry.

[276] Santarelli L., Saxe M., Gross C., Surget A., Battaglia F., Dulawa S., Weisstaub N., Lee J., Duman R., Arancio O. (2003). Requirement of Hippocampal Neurogenesis for the Behavioral Effects of Antidepressants. Science.

[277] Malberg J.E., Eisch A.J., Nestler E.J., Duman R.S. (2000). Chronic Antidepressant Treatment Increases Neurogenesis in Adult Rat Hippocampus. J. Neurosci..

[278] McEwen B.S., Magarinos A.M. (1997). Stress Effects on Morphology and Function of the Hippocampus. Ann. N. Y. Acad. Sci..

[279] Joëls M. (2007). Role of Corticosteroid Hormones in the Dentate Gyrus. Prog. Brain Res..

[280] Vilar M., Mira H. (2016). Regulation of Neurogenesis by Neurotrophins during Adulthood: Expected and Unexpected Roles. Front. Neurosci..

[281] Giuliani D., Zaffe D., Ottani A., Spaccapelo L., Galantucci M., Minutoli L., Bitto A., Irrera N., Contri M., Altavilla D. (2011). Treatment of Cerebral Ischemia with Melanocortins Acting at MC4 Receptors Induces Marked Neurogenesis and Long-Lasting Functional Recovery. Acta Neuropathol..

[282] Spaccapelo L., Galantucci M., Neri L., Contri M., Pizzala R., D’Amico R., Ottani A., Sandrini M., Zaffe D., Giuliani D. (2013). Up-Regulation of the Canonical Wnt-3A and Sonic Hedgehog Signaling Underlies Melanocortin-Induced Neurogenesis after Cerebral Ischemia. Eur. J. Pharmacol..

[283] Giuliani D., Neri L., Canalini F., Calevro A., Ottani A., Vandini E., Sena P., Zaffe D., Guarini S. (2015). NDP-α-MSH Induces Intense Neurogenesis and Cognitive Recovery in Alzheimer Transgenic Mice through Activation of Melanocortin MC4 Receptors. Mol. Cell Neurosci..

[284] Zhang Y., Wang J., Zhang D., Lu Z., Man J. (2020). Effects of RO27-3225 on Neurogenesis, PDGFRβ+ Cells and Neuroinflammation after Cerebral Infarction. Int. Immunopharmacol..

[285] Trullas R., Skolnick P. (1990). Functional Antagonists at the NMDA Receptor Complex Exhibit Antidepressant Actions. Eur. J. Pharmacol..

[286] McGrath T., Baskerville R., Rogero M., Castell L. (2022). Emerging Evidence for the Widespread Role of Glutamatergic Dysfunction in Neuropsychiatric Diseases. Nutrients.

[287] Suzuki A., Hara H., Kimura H. (2023). Role of the AMPA Receptor in Antidepressant Effects of Ketamine and Potential of AMPA Receptor Potentiators as a Novel Antidepressant. Neuropharmacology.

[288] Trifiletti R.R., Pranzatelli M.R. (1992). ACTH Binds to [3H]MK-801-Labelled Rat Hippocampal NMDA Receptors. Eur. J. Pharmacol..

[289] de Barioglio S.R., Brito M.I. (1996). Effect of Alpha-MSH upon Cyclic AMP Levels Induced by the Glutamatergic Agonists NMDA, Quisqualic Acid, and Kainic Acid. Peptides.

[290] Vasileva E.V., Kondrakhin E.A., Abdullina A.A., Salimov R.M., Kovalev G.I. (2020). Predominance of Nootropic or Anxiolytic Effects of Selank, Semax, and Noopept Peptides Depending on the Route of Administration to BALB/c and C57BL/6 Mice. Neurochem. J..

[291] Grigoriev V.V., Andreeva L.A., Zamoyski V.L., Shevchenko V.P., Bachurin S.O., Myasoedov N.F. (2015). The Action of the Peptide Drug Semax on the Currents of AMPA Receptors of Rat Cerebellar Purkinje Cells. Dokl. Biochem. Biophys..

[292] Shen Y., Fu W.-Y., Cheng E.Y.L., Fu A.K.Y., Ip N.Y. (2013). Melanocortin-4 Receptor Regulates Hippocampal Synaptic Plasticity through a Protein Kinase A-Dependent Mechanism. J. Neurosci..

[293] Lim B.K., Huang K.W., Grueter B.A., Rothwell P.E., Malenka R.C. (2012). Anhedonia Requires MC4R-Mediated Synaptic Adaptations in Nucleus Accumbens. Nature.

[294] Yao N., Skiteva O., Zhang X., Svenningsson P., Chergui K. (2018). Ketamine and Its Metabolite (2R,6R)-Hydroxynorketamine Induce Lasting Alterations in Glutamatergic Synaptic Plasticity in the Mesolimbic Circuit. Mol. Psychiatry.

[295] Bright U., Akirav I. (2022). Modulation of Endocannabinoid System Components in Depression: Pre-Clinical and Clinical Evidence. Int. J. Mol. Sci..

[296] Rana T., Behl T., Sehgal A., Mehta V., Singh S., Kumar R., Bungau S. (2021). Integrating Endocannabinoid Signalling In Depression. J. Mol. Neurosci..

[297] Yong Y., Cakir I., Lining Pan P., Biddinger J.E., Bluett R.J., Mackie K., Bingham N., Patel S., Ghamari-Langroudi M. (2021). Endogenous Cannabinoids Are Required for MC4R-Mediated Control of Energy Homeostasis. Proc. Natl. Acad. Sci. USA.

[298] Micale V., Drago F. (2018). Endocannabinoid System, Stress and HPA Axis. Eur. J. Pharmacol..

[299] Matias I., Vergoni A.V., Petrosino S., Ottani A., Pocai A., Bertolini A., Di Marzo V. (2008). Regulation of Hypothalamic Endocannabinoid Levels by Neuropeptides and Hormones Involved in Food Intake and Metabolism: Insulin and Melanocortins. Neuropharmacology.

[300] Maes M., DeJonckheere C., Vandervorst C., Schotte C., Cosyns P., Raus J., Suy E. (1991). Abnormal Pituitary Function during Melancholia: Reduced α-Melanocyte-Stimulating Hormone Secretion and Increased Intact ACTH Non-Suppression. J. Affect. Disord..

[301] Hidese S., Yoshida F., Ishida I., Matsuo J., Hattori K., Kunugi H. (2022). Plasma Neuropeptide Levels in Patients with Schizophrenia, Bipolar Disorder, or Major Depressive Disorder and Healthy Controls: A Multiplex Immunoassay Study. Neuropsychopharmacol. Rep..

[302] Berrettini W.H., Nurnberger J.I., Chan J.S.D., Chrousos G.P., Gaspar L., Gold P.W., Seidah N.G., Simmons-Alling S., Goldin L.R., Chrétien M. (1985). Pro-Opiomelanocortin-Related Peptides in Cerebrospinal Fluid: A Study of Manic-Depressive Disorder. Psychiatry Res..

[303] Sandman C. (1975). Enhancement of Attention in Man with ACTH/MSH 4–10. Physiol. Behav..

[304] Wu G.-S., Luo H.-R., Dong C., Mastronardi C., Licinio J., Wong M.-L. (2011). Sequence Polymorphisms of MC1R Gene and Their Association with Depression and Antidepressant Response. Psychiatr. Genet..

[305] Amin M., Ott J., Wu R., Postolache T.T., Gragnoli C. (2022). Implication of Melanocortin Receptor Genes in the Familial Comorbidity of Type 2 Diabetes and Depression. Int. J. Mol. Sci..

[306] Milaneschi Y., Simmons W.K., van Rossum E.F.C., Penninx B.W. (2019). Depression and Obesity: Evidence of Shared Biological Mechanisms. Mol. Psychiatry.

[307] Fulton S., Décarie-Spain L., Fioramonti X., Guiard B., Nakajima S. (2022). The Menace of Obesity to Depression and Anxiety Prevalence. Trends Endocrinol. Metab..

[308] Baldini G., Phelan K.D. (2019). The Melanocortin Pathway and Control of Appetite-Progress and Therapeutic Implications. J. Endocrinol..

[309] Kühnen P., Krude H., Biebermann H. (2019). Melanocortin-4 Receptor Signalling: Importance for Weight Regulation and Obesity Treatment. Trends Mol. Med..

[310] Yang Y., Xu Y. (2020). The Central Melanocortin System and Human Obesity. J. Mol. Cell Biol..

[311] Yeo G.S.H., Chao D.H.M., Siegert A.-M., Koerperich Z.M., Ericson M.D., Simonds S.E., Larson C.M., Luquet S., Clarke I., Sharma S. (2021). The Melanocortin Pathway and Energy Homeostasis: From Discovery to Obesity Therapy. Mol. Metab..

[312] Kokare D.M., Dandekar M.P., Singru P.S., Gupta G.L., Subhedar N.K. (2010). Involvement of α-MSH in the Social Isolation Induced Anxiety- and Depression-like Behaviors in Rat. Neuropharmacology.

[313] Chaki S., Hirota S., Funakoshi T., Suzuki Y., Suetake S., Okubo T., Ishii T., Nakazato A., Okuyama S. (2003). Anxiolytic-Like and Antidepressant-Like Activities of MCL0129 (1-[( *S* )-2-(4-Fluorophenyl)-2-(4-Isopropylpiperadin-1-Yl)Ethyl]-4-[4-(2-Methoxynaphthalen-1-Yl)Butyl]Piperazine), a Novel and Potent Nonpeptide Antagonist of the Melanocortin-4 Receptor. J. Pharmacol. Exp. Ther..

[314] Chaki S., Oshida Y., Ogawa S., Funakoshi T., Shimazaki T., Okubo T., Nakazato A., Okuyama S. (2005). MCL0042: A Nonpeptidic MC4 Receptor Antagonist and Serotonin Reuptake Inhibitor with Anxiolytic- and Antidepressant-like Activity. Pharmacol. Biochem. Behav..

[315] Liu J., Garza J.C., Truong H.V., Henschel J., Zhang W., Lu X.-Y. (2007). The Melanocortinergic Pathway Is Rapidly Recruited by Emotional Stress and Contributes to Stress-Induced Anorexia and Anxiety-Like Behavior. Endocrinology.

[316] Liu J., Garza J.C., Li W., Lu X.-Y. (2013). Melanocortin-4 Receptor in the Medial Amygdala Regulates Emotional Stress-Induced Anxiety-like Behaviour, Anorexia and Corticosterone Secretion. Int. J. Neuropsychopharmacol..

[317] Serova L.I., Laukova M., Alaluf L.G., Sabban E.L. (2013). Intranasal Infusion of Melanocortin Receptor Four (MC4R) Antagonist to Rats Ameliorates Development of Depression and Anxiety Related Symptoms Induced by Single Prolonged Stress. Behav. Brain Res..

[318] Sabban E.L., Serova L.I., Alaluf L.G., Laukova M., Peddu C. (2015). Comparative Effects of Intranasal Neuropeptide Y and HS014 in Preventing Anxiety and Depressive-like Behavior Elicited by Single Prolonged Stress. Behav. Brain Res..

[319] Chaki S., Okuyama S. (2005). Involvement of Melanocortin-4 Receptor in Anxiety and Depression. Peptides.

[320] Chaki S., Ogawa S., Toda Y., Funakoshi T., Okuyama S. (2003). Involvement of the Melanocortin MC4 Receptor in Stress-Related Behavior in Rodents. Eur. J. Pharmacol..

[321] Kokare D.M., Chopde C.T., Subhedar N.K. (2006). Participation of α-Melanocyte Stimulating Hormone in Ethanol-Induced Anxiolysis and Withdrawal Anxiety in Rats. Neuropharmacology.

[322] Corda M.G., Orlandi M., Fratta W. (1990). Proconflict Effect of ACTH1−24: Interaction with Benzodiazepines. Pharmacol. Biochem. Behav..

[323] Gonzalez M.I., Vaziri S., Wilson C.A. (1996). Behavioral Effects of α-MSH and MCH after Central Administration in the Female Rat. Peptides.

[324] Bruschetta G., Jin S., Liu Z.-W., Kim J.D., Diano S. (2020). MC4R Signaling in Dorsal Raphe Nucleus Controls Feeding, Anxiety, and Depression. Cell Rep..

[325] Cragnolini A.B., Schiöth H.B., Scimonelli T.N. (2006). Anxiety-like Behavior Induced by IL-1β Is Modulated by α-MSH through Central Melanocortin-4 Receptors. Peptides.

[326] Goyal S., Kokare D., Chopde C., Subhedar N. (2006). Alpha-Melanocyte Stimulating Hormone Antagonizes Antidepressant-like Effect of Neuropeptide Y in Porsolt’s Test in Rats. Pharmacol. Biochem. Behav..

[327] Deak T., Bellamy C., D’Agostino L.G., Rosanoff M., McElderry N.K., Bordner K.A. (2005). Behavioral Responses during the Forced Swim Test Are Not Affected by Anti-Inflammatory Agents or Acute Illness Induced by Lipopolysaccharide. Behav. Brain Res..

[328] Kastin A.J., Scollan E.L., Ehrensing R.H., Schally A.V., Coy D.H. (1978). Enkephalin and Other Peptides Reduce Passiveness. Pharmacol. Biochem. Behav..

[329] Vorvul A.O., Bobyntsev I.I., Medvedeva O.A., Mukhina A.Y., Svishcheva M.V., Azarova I.E., Andreeva L.A., Myasoedov N.F. (2022). ACTH(6-9)-Pro-Gly-Pro Ameliorates Anxiety-like and Depressive-like Behaviour and Gut Mucosal Microbiota Composition in Rats under Conditions of Chronic Restraint Stress. Neuropeptides.

[330] Markov D.D., Yatsenko K.A., Inozemtseva L.S., Grivennikov I.A., Myasoedov N.F., Dolotov O.V. (2017). Systemic N-Terminal Fragments of Adrenocorticotropin Reduce Inflammation- and Stress-Induced Anhedonia in Rats. Psychoneuroendocrinology.

[331] Kitamura Y., Araki H., Gomita Y. (2002). Influence of ACTH on the Effects of Imipramine, Desipramine and Lithium on Duration of Immobility of Rats in the Forced Swim Test. Pharmacol. Biochem. Behav..

[332] Kitamura Y., Akiyama K., Kitagawa K., Shibata K., Kawasaki H., Suemaru K., Araki H., Sendo T., Gomita Y. (2008). Chronic Coadministration of Carbamazepine Together with Imipramine Produces Antidepressant-like Effects in an ACTH-Induced Animal Model of Treatment–Resistant Depression: Involvement of 5-HT2A Receptors?. Pharmacol. Biochem. Behav..

[333] Walker A.J., Burnett S.A., Hasebe K., McGillivray J.A., Gray L.J., McGee S.L., Walder K., Berk M., Tye S.J. (2013). Chronic Adrenocorticotrophic Hormone Treatment Alters Tricyclic Antidepressant Efficacy and Prefrontal Monoamine Tissue Levels. Behav. Brain Res..

[334] Churruca I., Portillo M.P., Casis L., Gutiérrez A., Macarulla M.T., Echevarría E. (2008). Effects of Fluoxetine Administration on Hypothalamic Melanocortin System in Obese Zucker Rats. Neuropeptides.

[335] Baker R.A., Herkenham M., Brady L.S. (1996). Effects of Long-Term Treatment with Antidepressant Drugs on Proopiomelanocortin and Neuropeptide Y MRNA Expression in the Hypothalamic Arcuate Nucleus of Rats. J. Neuroendocrinol..

[336] Ortuño M.J., Schneeberger M., Ilanges A., Marchildon F., Pellegrino K., Friedman J.M., Ducy P. (2021). Melanocortin 4 Receptor Stimulation Prevents Antidepressant-Associated Weight Gain in Mice Caused by Long-Term Fluoxetine Exposure. J. Clin. Investig..

[337] van der Klaauw A.A., Keogh J.M., Henning E., Stephenson C., Kelway S., Trowse V.M., Subramanian N., O’Rahilly S., Fletcher P.C., Farooqi I.S. (2016). Divergent Effects of Central Melanocortin Signalling on Fat and Sucrose Preference in Humans. Nat. Commun..

[338] Panaro B.L., Cone R.D. (2013). Melanocortin-4 Receptor Mutations Paradoxically Reduce Preference for Palatable Foods. Proc. Natl. Acad. Sci. USA.

[339] Lippert R.N., Ellacott K.L.J., Cone R.D. (2014). Gender-Specific Roles for the Melanocortin-3 Receptor in the Regulation of the Mesolimbic Dopamine System in Mice. Endocrinology.

[340] Eliason N.L., Martin L., Low M.J., Sharpe A.L. (2022). Melanocortin Receptor Agonist Melanotan-II Microinjected in the Nucleus Accumbens Decreases Appetitive and Consumptive Responding for Food. Neuropeptides.

[341] Shanmugarajah L., Dunigan A.I., Frantz K.J., Roseberry A.G. (2017). Altered Sucrose Self-Administration Following Injection of Melanocortin Receptor Agonists and Antagonists into the Ventral Tegmental Area. Psychopharmacology.

[342] Yen H.-H., Roseberry A.G. (2015). Decreased Consumption of Rewarding Sucrose Solutions after Injection of Melanocortins into the Ventral Tegmental Area of Rats. Psychopharmacology.

[343] Pandit R., Omrani A., Luijendijk M.C.M., de Vrind V.A.J., Van Rozen A.J., Ophuis R.J.A.O., Garner K., Kallo I., Ghanem A., Liposits Z. (2016). Melanocortin 3 Receptor Signaling in Midbrain Dopamine Neurons Increases the Motivation for Food Reward. Neuropsychopharmacology.

[344] Allen A.T., Heaton E.C., Shapiro L.P., Butkovich L.M., Yount S.T., Davies R.A., Li D.C., Swanson A.M., Gourley S.L. (2022). Inter-Individual Variability Amplified through Breeding Reveals Control of Reward-Related Action Strategies by Melanocortin-4 Receptor in the Dorsomedial Striatum. Commun. Biol..

[345] Pandit R., van der Zwaal E.M., Luijendijk M.C.M., Brans M.A.D., van Rozen A.J., Oude Ophuis R.J.A., Vanderschuren L.J.M.J., Adan R.A.H., la Fleur S.E. (2015). Central Melanocortins Regulate the Motivation for Sucrose Reward. PLoS ONE.

[346] Figlewicz D.P., Jay J.L., Acheson M.A., Magrisso I.J., West C.H., Zavosh A., Benoit S.C., Davis J.F. (2013). Moderate High Fat Diet Increases Sucrose Self-Administration in Young Rats. Appetite.

[347] Cabeza de Vaca S., Hao J., Afroz T., Krahne L.L., Carr K.D. (2005). Feeding, Body Weight, and Sensitivity to Non-Ingestive Reward Stimuli during and after 12-Day Continuous Central Infusions of Melanocortin Receptor Ligands. Peptides.

[348] Grieb Z.A., Cross E.A., Albers H.E. (2022). Alpha-Melanocyte-Stimulating Hormone (AMSH) Modulates the Rewarding Properties of Social Interactions in an Oxytocin Receptor-Dependent Manner in Syrian Hamsters (Mesocricetus Auratus). Physiol. Behav..

[349] Klawonn A.M., Fritz M., Nilsson A., Bonaventura J., Shionoya K., Mirrasekhian E., Karlsson U., Jaarola M., Granseth B., Blomqvist A. (2018). Motivational Valence Is Determined by Striatal Melanocortin 4 Receptors. J. Clin. Investig..

[350] Qu N., He Y., Wang C., Xu P., Yang Y., Cai X., Liu H., Yu K., Pei Z., Hyseni I. (2020). A POMC-Originated Circuit Regulates Stress-Induced Hypophagia, Depression, and Anhedonia. Mol. Psychiatry.

[351] Legrand R., Lucas N., Breton J., Déchelotte P., Fetissov S.O. (2015). Dopamine Release in the Lateral Hypothalamus Is Stimulated by α-MSH in Both the Anticipatory and Consummatory Phases of Feeding. Psychoneuroendocrinology.

[352] Rudman D., Hollins B.M., Kutner M.H., Moffitt S.D., Lynn M.J. (1983). Three Types of Alpha-Melanocyte-Stimulating Hormone: Bioactivities and Half-Lives. Am. J. Physiol. Metab..

[353] Di L. (2015). Strategic Approaches to Optimizing Peptide ADME Properties. AAPS J..

[354] Lamers C. (2022). Overcoming the Shortcomings of Peptide-Based Therapeutics. Futur. Drug Discov..

[355] Wilson J. (1988). Low Permeability of the Blood-Brain Barrier to Nanomolar Concentrations of Immunoreactive Alpha-Melanotropin. Psychopharmacology.

[356] Kiss J.Z., Mezey E., Cassell M.D., Williams T.H., Mueller G.P., O’Donohue T.L., Palkovits M. (1985). Topographical Distribution of Pro-Opiomelanocortin-Derived Peptides (ACTH/β-END/α-MSH) in the Rat Median Eminence. Brain Res..

[357] Van Houten M., Khan M.N., Walsh R.J., Baquiran G.B., Renaud L.P., Bourque C., Sgro S., Gauthier S., Chretien M., Posner B.I. (1985). NH2-Terminal Specificity and Axonal Localization of Adrenocorticotropin Binding Sites in Rat Median Eminence. Proc. Natl. Acad. Sci. USA.

[358] Tatro J.B. (1990). Melanotropin Receptors in the Brain Are Differentially Distributed and Recognize Both Corticotropin and α-Melanocyte Stimulating Hormone. Brain Res..

[359] Tatro J.B. (1993). Brain Receptors for Central and Peripheral Melanotropins. Ann. N. Y. Acad. Sci..

[360] Potaman V.N., Antonova L.V., Dubynin V.A., Zaitzev D.A., Kamensky A.A., Myasoedov N.F., Nezavibatko V.N. (1991). Entry of the Synthetic ACTH(4–10) Analogue into the Rat Brain Following Intravenous Injection. Neurosci. Lett..

[361] Shevchenko K.V., Nagaev I.Y., Alfeeva L.Y., Andreeva L.A., Kamenskii A.A., Levitskaya N.G., Shevchenko V.P., Grivennikova I.A., Myasoedov N.F. (2006). Kinetics of Semax Penetration into the Brain and Blood of Rats after Its Intranasal Administration. Russ. J. Bioorganic Chem..

[362] Trivedi P., Jiang M., Tamvakopoulos C.C., Shen X., Yu H., Mock S., Fenyk-Melody J., Van der Ploeg L.H., Guan X.-M. (2003). Exploring the Site of Anorectic Action of Peripherally Administered Synthetic Melanocortin Peptide MT-II in Rats. Brain Res..

[363] Fries G.R., Saldana V.A., Finnstein J., Rein T. (2023). Molecular Pathways of Major Depressive Disorder Converge on the Synapse. Mol. Psychiatry.

